# tRNA Modifying Enzymes, NSUN2 and METTL1, Determine Sensitivity to 5-Fluorouracil in HeLa Cells

**DOI:** 10.1371/journal.pgen.1004639

**Published:** 2014-09-18

**Authors:** Mayumi Okamoto, Mamoru Fujiwara, Masato Hori, Kaoru Okada, Futoshi Yazama, Hiroaki Konishi, Yegui Xiao, Guangying Qi, Fumio Shimamoto, Takahide Ota, Achim Temme, Masaaki Tatsuka

**Affiliations:** 1 Department of Life Sciences, Faculty of Life and Environmental Sciences, Prefectural University of Hiroshima, Shoubara, Hiroshima, Japan; 2 Department of Management Information Systems, Faculty of Management and Information System, Prefectural University of Hiroshima, Minami-ku, Hiroshima, Japan; 3 Department of Health Sciences, Faculty of Human Culture and Science, Prefectural University of Hiroshima, Minami-ku, Hiroshima, Japan; 4 Department of Life Science, Medical Research Institute, Kanazawa Medical University, Uchinada, Ishikawa, Japan; 5 Department of Neurosurgery, University Hospital Carl Gustav Carus, Technical University Dresden, Dresden, Germany; University of Washington, United States of America

## Abstract

Nonessential tRNA modifications by methyltransferases are evolutionarily conserved and have been reported to stabilize mature tRNA molecules and prevent rapid tRNA decay (RTD). The tRNA modifying enzymes, NSUN2 and METTL1, are mammalian orthologs of yeast Trm4 and Trm8, which are required for protecting tRNA against RTD. A simultaneous overexpression of NSUN2 and METTL1 is widely observed among human cancers suggesting that targeting of both proteins provides a novel powerful strategy for cancer chemotherapy. Here, we show that combined knockdown of NSUN2 and METTL1 in HeLa cells drastically potentiate sensitivity of cells to 5-fluorouracil (5-FU) whereas heat stress of cells revealed no effects. Since NSUN2 and METTL1 are phosphorylated by Aurora-B and Akt, respectively, and their tRNA modifying activities are suppressed by phosphorylation, overexpression of constitutively dephosphorylated forms of both methyltransferases is able to suppress 5-FU sensitivity. Thus, NSUN2 and METTL1 are implicated in 5-FU sensitivity in HeLa cells. Interfering with methylation of tRNAs might provide a promising rationale to improve 5-FU chemotherapy of cancer.

## Author Summary

The cellular mechanisms for sensing and responding to stress on nucleic acid metabolism or to genotoxic stress are the fundamental and ancient evolutionary biological activities with conserved and diverse biological functions. In yeast, hypomodified mature tRNA species are rapidly decayed under heat stress by the RTD pathway. Yet, it has been shown that tRNA-specific methyltransferases Trm4 and Trm8 protect from tRNA decay. 5-FU, a pyrimidine analog used for cancer treatment, is generally known to act as a thymidylate synthase inhibitor although other ways for the mechanisms of action are suggested. We studied NSUN2 and METTL1, the human orthologs of Trm4 and Trm8 in yeast, and demonstrated that these RTD-related tRNA modifying enzymes are involved in 5-FU sensitivity in cervical cancer HeLa cells. We conclude that the evolutionarily conserved regulation of tRNA modifications is a potential mechanism of chemotherapy resistance in cancer cells.

## Introduction

5-Fluorouracil (5-FU) is a pyrimidine analog and is the most widely used chemotherapeutic agent for the treatment of a variety of solid cancers. Its mechanism of action has been attributed to the production of cytotoxic metabolites incorporated into RNA and DNA and inhibiting thymidylate synthase, finally leading to cell cycle arrest and apoptosis in cancer cells [1]. 5-FU is used against cancer for about 40 years and it is known that systemic administration of 5-FU might result in drug resistance of tumor cells. Furthermore, treatment regimens with increased dosage of 5-FU have been reported to cause severe side effects such as myelosuppression, mucositis, dermatitis and diarrhea., In order to address this dilemma, different strategies were pursued to improve outcomes for patients and to reduce side effects of 5-FU therapy [2]–[9]. However, also with current approaches, there is still a need to develop new compounds or novel strategies by which cancer cells are killed more effectively and more selectively [10]–[12].

Overexpression of tRNA-modifying enzymes NSUN2 and METTL1 is widely observed among human cancers [13]–[16]. NSUN2 (NOP2/Sun domain family, member 2), also known as SAKI (Substrate of AIM-1/Aurora kinase B), is a NOL1/NOP2/SUN domain-containing tRNA (cytosine-5-)-methyltransferase. It is phosphorylated at Ser139 by Aurora-B to inhibit its enzymatic activity during mitosis [17]. Trm4, a yeast *Saccharomyces cerevisiae* homologue of human NSUN2, participates in the nonessential modification of tRNA [18], [19], and a yeast mutant deficient in Trm4 shows no defect in cell growth and has normal sensitivities to various stresses [18], [19].

On the other hand, another tRNA modification enzyme Trm8, which is also nonessential and catalyzes tRNA 7-methylguanosine modification [20], acts together with Trm4 to stabilize tRNA under heat stress [21]. If tRNA modifications caused by Trm4 and Trm8 are defective, a rapid degradation of tRNA is induced under heat stress, resulting in the expression of heat-sensitive phenotype [21]. The tRNA surveillance system that monitors compromised tRNAs with no modification by Trm4 and Trm8 uses a rapid tRNA degradation (RTD) pathway to decay non-modified tRNAs, leading to cell death [21]–[23].

A human tRNA (guanine-N7-)-methyltransferase, a homologue of yeast Trm8, is known as METTL1 (methyltransferase like 1) [20], [24]. Whereas NSUN2 has been initially identified as a substrate of protein kinase (Aurora-B) in HeLa cells [17], METTL1 has been initially identified as a substrate of Akt/protein kinase Bα (PKBα) in HeLa cells [13]. Interestingly, phosphorylated METTL1 at Ser27 by Akt is also enzymatically inactive [13]. The fact that both tRNA methyltransferases are evolutionally conserved suggests a similar tRNA surveillance system including Trm4 and Trm8 in human cells. Furthermore, the observation that the cytotoxic effect of 5-FU in yeast is enhanced by heat stress in a *trm8* mutant strain [25] leads us to the hypothesis that nonessential tRNA modifications catalyzed by NSUN2 and METTL1 impacts the efficiency of 5-FU treatment in human cancer cells.

Here, we provide evidence that tRNA methyltransferases, NSUN2 and METTL1, strongly influences 5-FU sensitivity in human cancer cells. Therefore, targeting these methyltransferases might represent a promising rationale to improve 5-FU-treatment of tumors and to reduce 5-FU-related side effects in patients.

## Results

### NSUN2 did not affect cell growth

NSUN2 (SAKI) has been reported to be overexpressed and with gain in gene copy-number in various of human cancers [15]. Furthermore, NSUN2 has been implicated in myc-induced proliferation [26]. In line with these observations, the siRNA-mediated knockdown of NSUN2 negatively affects cancer cell growth [14] and homozygous knockout of the *NSUN2* gene locus causes delayed cell growth in bulge stem cells [27]. However, in our previous studies, NSUN2 expression was not altered during the cell cycle of HeLa cervix carcinoma cells [17]. When we investigated normal human diploid fibroblasts, NSUN2 expression was found to be very low compared with HeLa cells and again NSUN2 was not differentially expresses during the cell cycle [17].

In initial studies we sought to analyze the impact of increased or decreased NSUN2 expression on the growth properties of HeLa cells. We therefore utilized cell lines clonally derived from stable transfectants described previously [17]. These studies indicated that there was a difference in the growth properties that arise as a result of heterogeneity among clones although we found that NSUN2 did not alter the growth properties of HeLa cells both onto plastic dish culture and in semisolid agar culture (Figure S1). Subsequently, we pooled cells from five independent clones for further experiments and examined expression levels of NSUN2 and METTL1. We then generated Xpress-NSUN2-overexpressing HeLa cells as well as NSUN2 knockdown cells, the latter by using an shRNA targeting the 5′-UTR of NSUN2 mRNA. Successively we tested cell growth both onto plastic dish culture and in semisolid agar culture. The data clearly indicated that NSUN2 is related to neither cell multiplication nor cancerous cell growth (Figure S2 and S3).

### Co-overexpression of NSUN2 and METTL1 confers a protective effect of 5-FU-induced cytotoxicity

**Figure 1:**
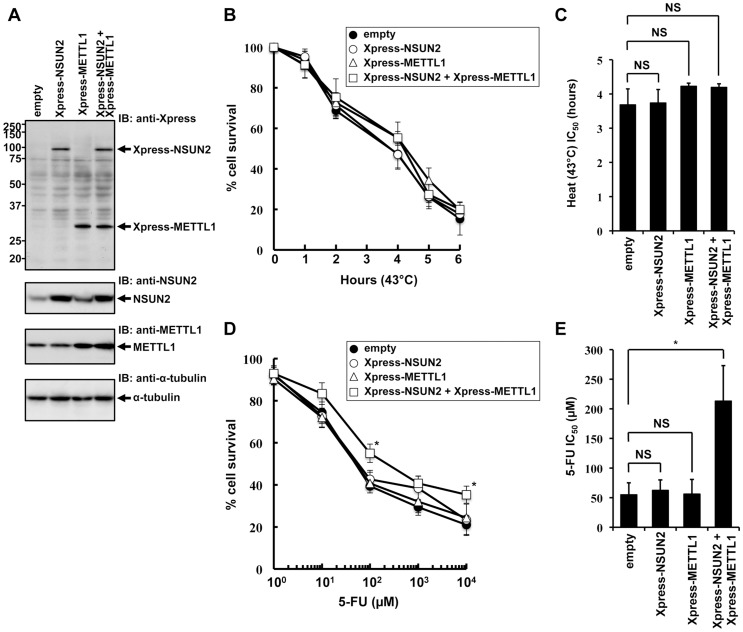
Effects of increased expression of NSUN2 and METTL1 on 5-FU-treatment and heat stress and 5-FU. (A) Immunoblot analysis of proteins from Xpress-NSUN2-overexpressing cells (lane *Xpress-NSUN2*), Xpress-METTL1-overexpressing cells (lane *Xpress-METTL1*), Xpress-NSUN2 and Xpress-METTL1-co-overexpressing cells (lane *Xpress-NSUN2 + Xpress-METTL1*), and control vector-transfected cells (lane *empty*) with anti-Xpress, anti-NSUN2, anti-METTL1 and anti-α-tubulin antibodies. (B) Time-dependent cell survival in response to heat stress in Xpress-NSUN2-overexpressing cells (*open circle*), Xpress-METTL1-overexpressing cells (*open triangle*), Xpress-NSUN2 and Xpress-METTL1-co-overexpressing cells (*open square*), and control vector-transfected cells (*closed circle*) with the MTT viability assay. There was no significant difference in survival of Xpress-NSUN2- or Xpress-METTL-1-overexpressing cells compared with control vector-transfected cells in all of each duration period. (C) Comparison of the average IC_50_ values for heat stress between Xpress-NSUN2- or Xpress-METTL-1-overexpressing cells and control vector-transfected cells. NS, not significant. (D) Dose-dependent cell survival in response to 5-FU in Xpress-NSUN2-overexpressing cells (*open circle*), Xpress-METTL1-overexpressing cells (*open triangle*), Xpress-NSUN2 and Xpress-METTL1-co-overexpressing cells (*open square*), and control vector-transfected cells (*closed circle*) with the MTT viability assay. *Significant difference compared with control vector-transfected cells (*P*<0.05). (E) Comparison of the average IC_50_ values for 5-FU between Xpress-NSUN2- and/or Xpress-METTL-1-overexpressing cells and control vector-transfected cells. Data throughout this study represent the mean ± SD for three independent experiments, and error bars represent the SD. Difference between values were analyzed using a two-tailed Welch's *t*-test. *P*-values of <0.05 were considered significant. NS, not significant. *Significant difference compared with control vector-transfected cells (*P*<0.05).

To further elucidate NSUN2 function in mammalian cells, we focused on mechanisms involved in tRNA methylation. NSUN2 is a mammalian homolog of yeast Trm4. In yeast system, Trm4-mediated tRNA modification is nonessential, but the additional knockout of Trm8, which is tRNA (guanine-N7-)-methyltransferase, under Trm4 knockout background leads to an unstable tRNA situation, resulting in a temperature-sensitive growth. Based on cooperative functions of Trm4 and Trm8 in yeast, we sought to analyze the effects of overexpressed NSUN2 and METTL1 in HeLa cells suffering heat stress. For this we used HeLa cell lines engineered to express NSUN2, METTL1 and both methyltransferases. The ectopic expression of the methyltransferases was confirmed by Western blot analysis as depicted in Figure 1A. Contrary to our expectations, overexpression of NSUN2 and METTL1 did not affect heat stress-induced cytotoxicity (Figure 1A, 1B and 1C).

Next, we sought to investigate whether overexpression of NSUN2 and METTL1 protects from 5-FU-induced cytotoxicity, since tRNA modifying enzymes have been implicated as *in vivo* targets for 5-FU in yeast [25]. Although we could not observe a protective effect after 5-FU-treatment in HeLa cells expressing NSUN2 or METTL1 alone, we revealed that combined overexpression of NSUN2 and METTL1 significantly protected HeLa cells from 5-FU-induced cell death (Figure 1D and 1E). Noteworthy, co-overexpression of NSUN2 and METTL1 did not affect cell proliferation growth in soft agar (Figure S4).

### Double knockdown of NSUN2 and METTL1 potentiated the cytotoxic effect of 5-FU

**Figure 2:**
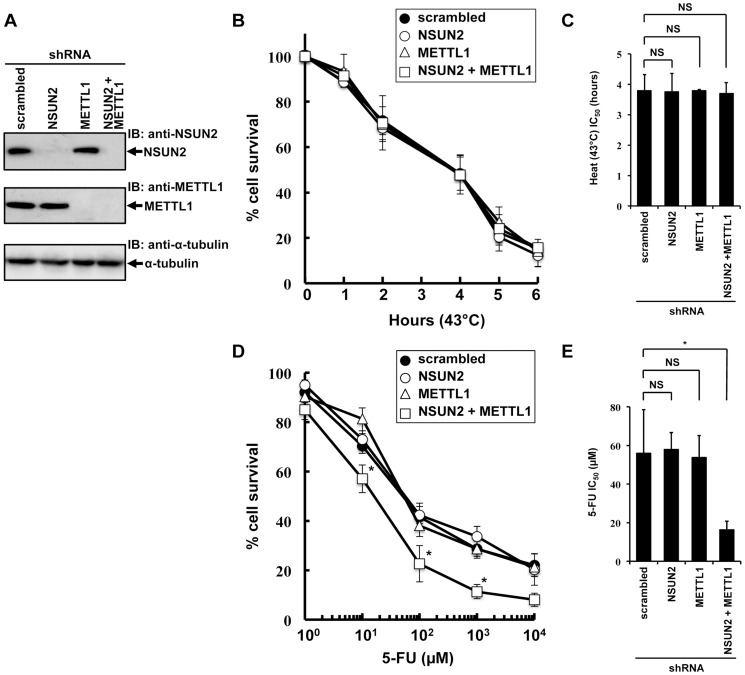
Effects of decreased NSUN2 and METTL1 expression on 5-FU-treatment and heat stress and 5-FU. (A) Immunoblot analysis of total proteins lysates and immunoprecipitated NSUN2 and METTL1 proteins from indicated NSUN2-, METTL1-, and double NSUN2/METTL1 knockdowns and control transfectant. As loading control the western blot was probed with an anti-α-tubulin antibody. (B) Time-dependent cell survival in response to heat stress in NSUN2 knockdown cells (*open circle*), METTL1 knockdown cells (*open triangle*), NSUN2 and METTL1 knockdown cells (*open square*), and control vector-transfected cells (*closed circle*) with the MTT viability assay. There was no significant difference in survival of NSUN2 knockdown cells, METTL-1 knockdown cells, or NSUN2 and METTL1 knockdown cells compared with control vector-transfected cells in all of each duration period. (C) Comparison of the average IC_50_ values for heat stress between NSUN2 knockdown cells, METTL-1 knockdown cells, or NSUN2 and METTL1 knockdown cells and control vector-transfected cells. NS, not significant. (D) Dose-dependent cell survival in response to 5-FU in NSUN2 knockdown cells (*open circle*), METTL1 knockdown cells (*open triangle*), NSUN2 and METTL1 knockdown cells (*open square*), and control vector-transfected cells (*closed circle*) with the MTT viability assay. *Significant difference compared with control vector-transfected cells (*P*<0.05). (E) Comparison of the average IC_50_ values for 5-FU between NSUN2 knockdown cells, METTL-1 knockdown cells, or NSUN2 and METTL1 knockdown cells and control vector-transfected cells. NS, not significant. *Significant difference compared with control vector-transfected cells (*P*<0.05).

To examine the effects of double knockdown of NSUN2 and METTL1 on heat stress-induced cytotoxicity and 5-FU-induced cytotoxicity, we established stable HeLa cells with knockdown of NSUN2, METTL1, or NSUN2 and METTL1. As depicted in Figure 2A we fully knocked down NSUN2, METTL1 protein expression and were also able to achieve an effective NSUN2/METTL1 double knockdown.

Interestingly, the knockdown of NSUN2 did not affect cell multiplication and cancerous cell growth (Figure S5). Also, we noted that knockdown of METTL1 and double knockdown of NSUN2 and METTL1 did not affect cell multiplication and cancerous cell growth (Figure S5).

Since it was of special interest whether the knockdown of NSUN2, METTL1 and double knockdown of NSUN2 and METTL1 results in a temperature-sensitive growth phenotype as reported in yeast we incubated the cells at 43°C for a time period of 1 h to 6 h. Contrary to our expectations, the double knockdown of NSUN2 and METTL1 did not enhance heat stress-induced cytotoxicity (Figure 2B and 2C).

Subsequently we examined whether the double knockdown of NSUN2 and METTL1 affects 5-FU-induced cytotoxicity. Consistent with our observation of NSUN2 and METTL1 overexpression experiments, which shows protective effects of tRNA methyltransferases, we revealed that double knockdown of NSUN2 and METTL1 significantly sensitized HeLa cells to 5-FU treatment (Figure 2D and 2E).

### Double knockdown of NSUN2 and METTL1 did not affect cisplatin- and paclitaxel-induced cytotoxicity

**Figure 3:**
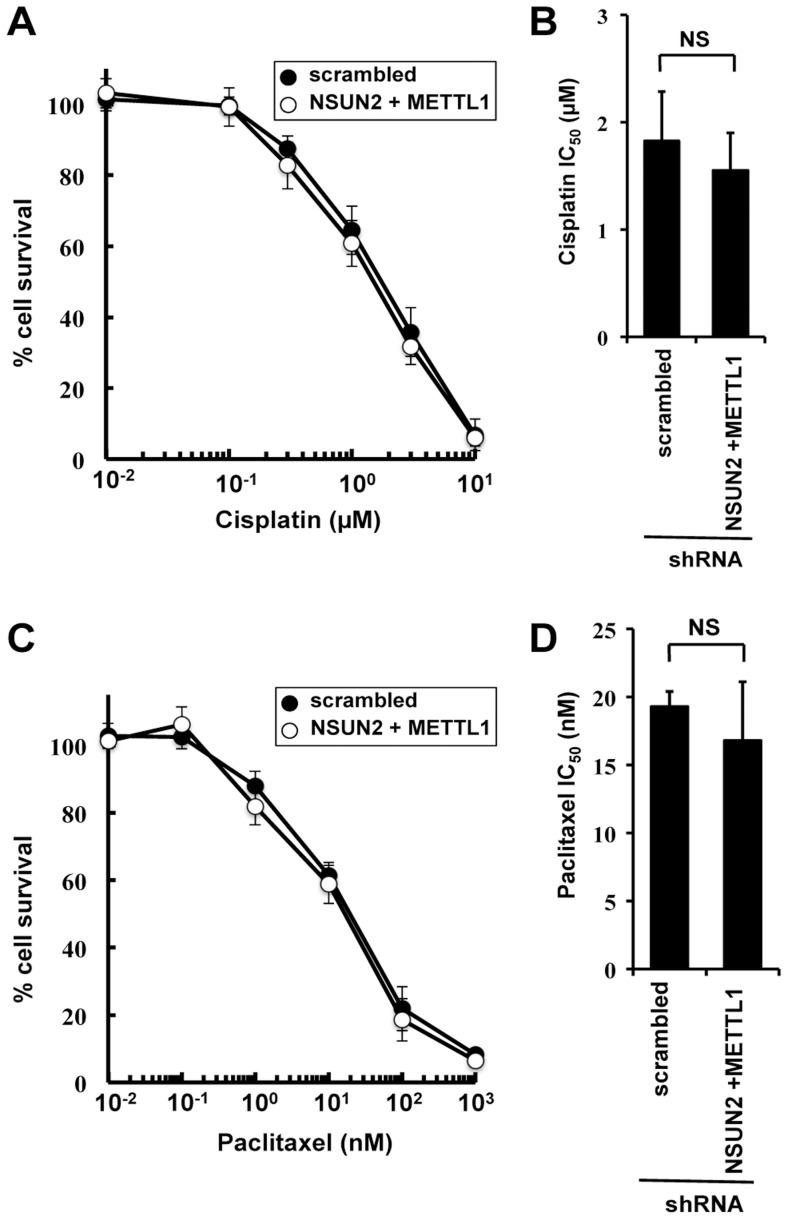
Effect of cisplatin and paclitaxel treatment in NSUN2 and METTL1 knockdown and double knockdown cells. (A) Dose-dependent cell survival in response to cisplatin in NSUN2 and METTL1 knockdown cells (*open circle*) and control vector-transfected cells (*closed circle*) with the MTT viability assay. There was no significant difference in survival of NSUN2 and METTL1 knockdown cells compared with control vector-transfected cells in all of each concentration. (B) Comparison of the average IC_50_ values for cisplatin between NSUN2 and METTL1 knockdown cells and control vector-transfected cells. NS, not significant. (C) Dose-dependent cell survival in response to paclitaxel in NSUN2 and METTL1 knockdown cells (*open circle*) and control vector-transfected cells (*closed circle*) with the MTT viability assay. There was no significant difference in survival of NSUN2 and METTL1 knockdown cells compared with control vector-transfected cells in all of each concentration. (D) Comparison of the average IC_50_ values for paclitaxel between NSUN2 and METTL1 knockdown cells and control vector-transfected cells. NS, not significant.

To examine the effects of double knockdown of NSUN2 and METTL1 on further chemotherapeutic agents other than 5-FU, we used cisplatin, a platinum-containing anti-cancer drug that binds to and causes crosslinking of DNA, and paclitaxel, a microtubule-stabilizing mitotic inhibitor. Contradictory to 5-FU treatment, both agents caused no detectable differences in induced cytotoxicity between control cells and double knockout (Figure 3).

### Double knockdown of NSUN2 and METTL1 caused increased sensitivity to 5-FU-induced cell death measured by colony formation assay

**Figure 4:**
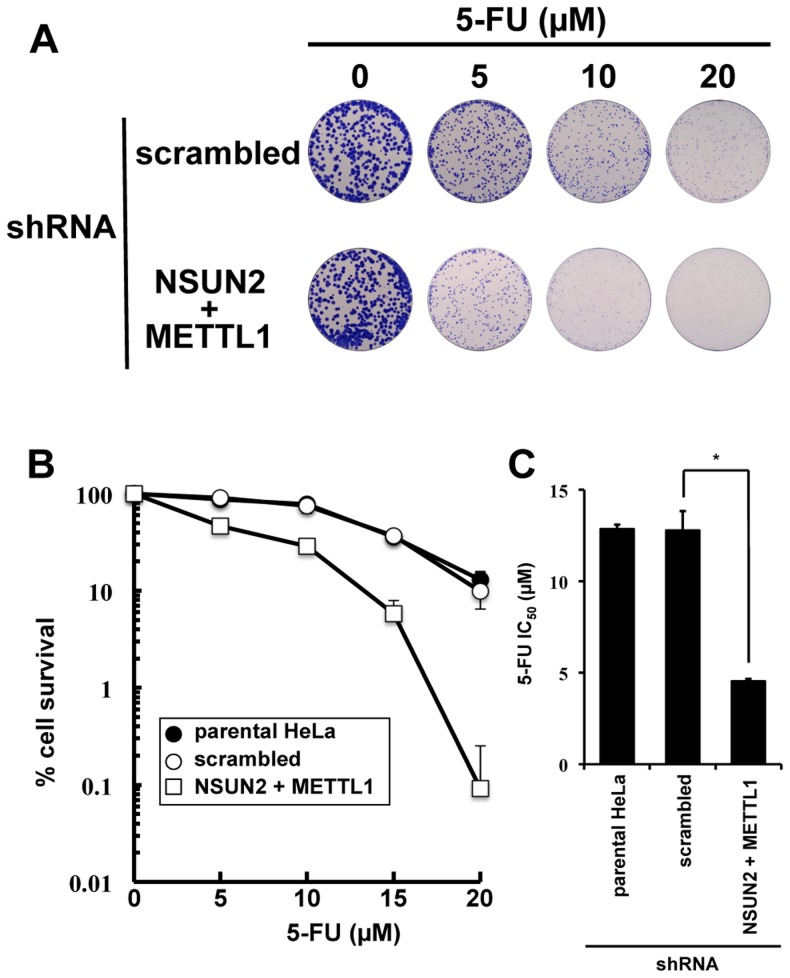
Colony formation assay showing synergistic effects of NSUN2 and METTL1 double knock down and 5-FU-treatment. (A) Depiction of colonies formed after 5-FU treatment at various concentrations on Giemsa-stained dishes. (B) Dose-dependent cell survival in response to 5-FU in NSUN2 and METTL1 knockdown cells (*open triangle*), the parent cells (*closed circle*), and control vector-transfected cells (*open circle*) in colony formation assay. (C) Comparison of the average IC_50_ values for 5-FU assessed by colony formation assay between NSUN2 and METTL1 knockdown cells and control vector-transfected cells. *Significant difference compared with control vector-transfected cells (*P*<0.05).

As shown in Figure 2, we found that double knockdown of NSUN2 and METTL1 potentiated the cytotoxic effect of 5-FU, measured by MTT assay. To confirm our results, colony formation assay, a method capable to essentially test every cell in the population for its ability to cell multiplication escaping cell reproductive death, was used to determine the effectiveness of 5-FU. The data revealed that double knockdown of NSUN2 and METTL1 caused increased sensitivity to 5-FU-treatment resulting in a dose-dependent and significant decreased clonal survival when compared to scrambled shRNA control (Figure 4). NSUN2 and METTL1 knockdown cells treated with 5–20 µM 5-FU showed a 60 - 68% decreased values of the average IC_50_ (4.54±0.12 µM) compared with those of the parent cells (12.85±0.25 µM) and control vector-transfected cells (12.79±1.05 µM) (Figure 4C).

### Double knockdown of NSUN2 and METTL1 caused rapid tRNA(Val^AAC^) degradation induced by 5-FU

**Figure 5:**
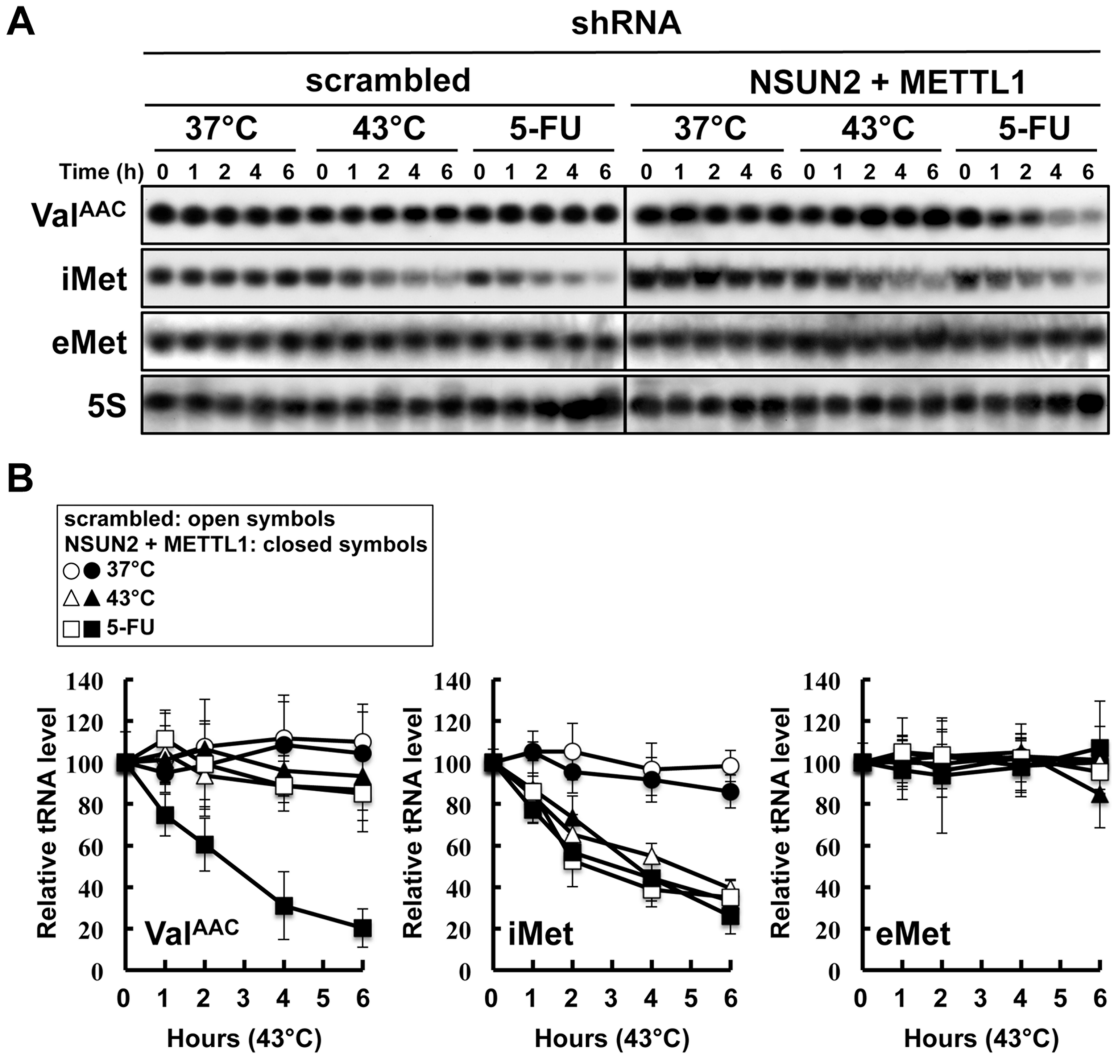
Degradation of tRNAs in HeLa cells treated with heat stress or 5-FU. (A) Northern blot analysis of tRNA(Val^AAC^), tRNA(iMet), tRNA(eMet), and 5S rRNA. NSUN2 and METTL1 knockdown cells and control vector-transfected cells were treated with 37°C (control), 43°C (heat stress), or 5-FU (7.69 mM) for indicated time. Total RNA (20 µg) was loaded and blotted. (B) Degradation profile of tRNA(Val^AAC^), tRNA(iMet), and tRNA(eMet) in NSUN2 and METTL1 knockdown cells (closed symbols) and control vector-transfected cells (open symbols). The cells were treated with 37°C (*circle*), 43°C (*triangle*), and 5-FU (*square*). 5S rRNA transcribed by RNA polymerase III was used as a control, and quantitative data were indicated.

In the yeast system, a double knockout of Trm4 and Trm8, the orthologs of human NSUN2 and METTL1, shows an increased sensitivity to heat stress, resulting in a rapid tRNA(Val^AAC^) degradation. A similar situation may therefore occur when exposing HeLa cells depleted of NSUN2 and METTL1 to 5-FU. To address this question we performed tRNA stability assays using HeLa cells with double knockdown of NSUN2 and METTL1 and as control HeLa cells transfected wit scramble shRNA. As shown in Figure 5, tRNA(Val^AAC^) was unstable in HeLa cells with double knockdown of NSUN2 and METTL1 when exposed to 5-FU whereas in control cells the amount of tRNA(Val^AAC^) remained stable. Interestingly, we monitored a rapid degradation of initiator tRNA^Met^ (tRNA(iMet)) in both control cells and knockdown cells when exposed to heat stress and even when exposed to 5-FU whereas the amounts of elongator tRNA^Met^ (tRNA(eMet)) molecules were not affected.

### Co-expression of a dephosphorylation-mimic NSUN2 and a dephosphorylation-mimic METTL1 conferred significant protection against 5-FU-induced cytotoxicity but phosphorylation-mimic forms did not

**Figure 6:**
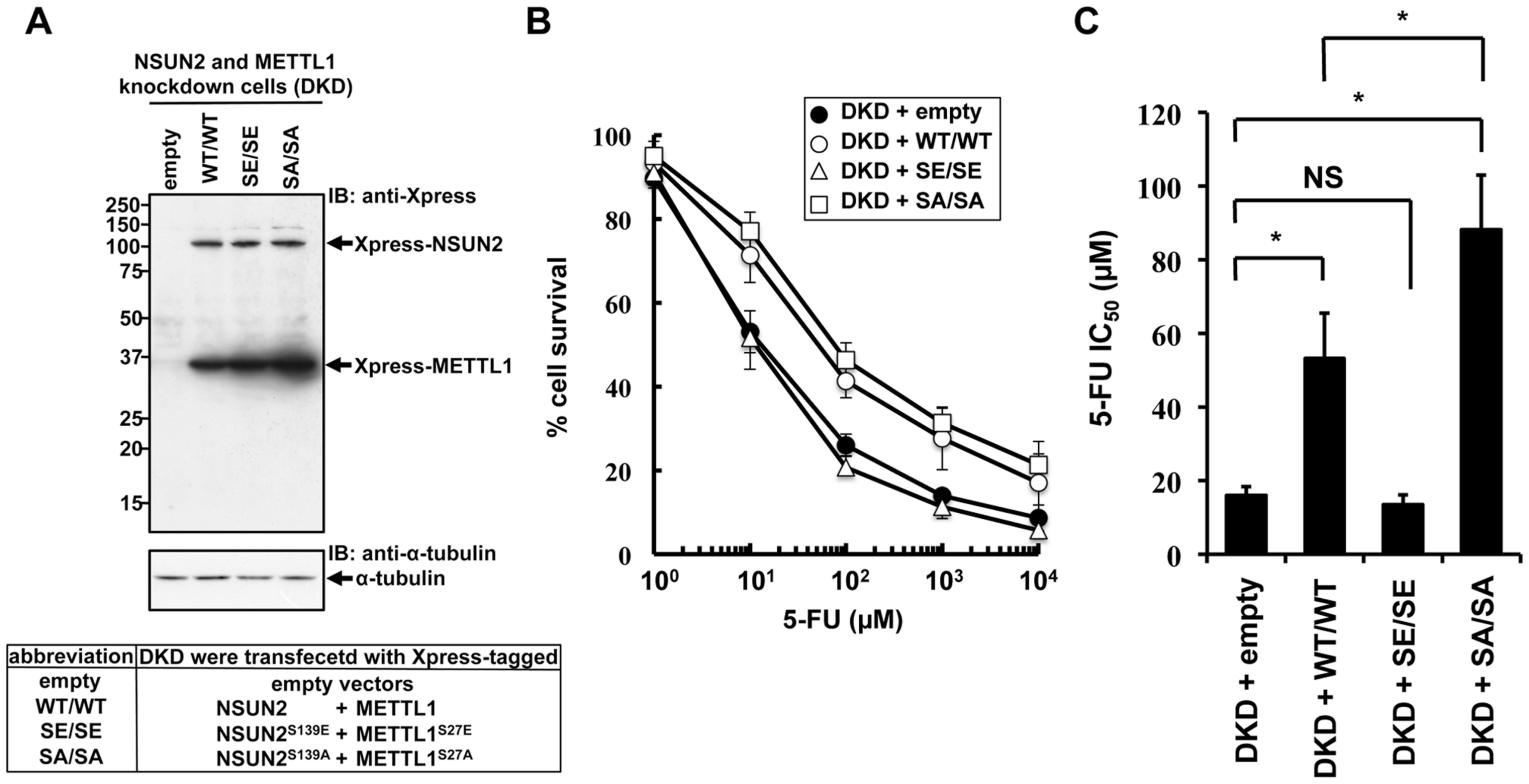
Effects of co-expression of NSUN2 and METTL1 on the cytotoxic effects of heat stress and 5-FU in NSUN2 and METTL1 knockdown cells. (A) Immunoblot analysis of proteins from NSUN2 and METTL1 knockdown cells co-expressing Xpress-tagged wild type NSUN2 and Xpress-tagged wild type METTL1 (lane *WT/WT*), NSUN2 and METTL1 knockdown cells co-expressing Xpress-tagged phosphorylation-mimic NSUN2 and Xpress-tagged phosphorylation-mimic METTL1 (lane *SE/SE*), NSUN2 and METTL1 knockdown cells co-expressing Xpress-tagged dephosphorylation-mimic NSUN2 and Xpress-tagged dephosphorylation-mimic METTL1 (lane *SA/SA*), and NSUN2 and METTL1 knockdown cells transfected with control vector (lane *empty*) with anti-Xpress and anti-α-tubulin antibodies. Five independent clones that co-expressed Xpress-NSUN2 and Xpress-METTL1 or were transfected with the empty vector, originating from UTR-targeting shRNA-mediated NSUN2 and METTL1 knockdown cells, were pooled and used as a stable transfectant. (B) Dose-dependent cell survival in response to 5-FU in NSUN2 and METTL1 knockdown cells co-expressing Xpress-tagged wild type NSUN2 and Xpress-tagged wild type METTL1 (*open circle, DKD + WT/WT*), NSUN2 and METTL1 knockdown cells co-expressing Xpress-tagged phosphorylation-mimic NSUN2 and Xpress-tagged phosphorylation-mimic METTL1 (*open triangle, DKD + SE/SE*), NSUN2 and METTL1 knockdown cells co-expressing Xpress-tagged dephosphorylation-mimic NSUN2 and Xpress-tagged dephosphorylation-mimic METTL1 (*open square, DKD + SA/SA*), and NSUN2 and METTL1 knockdown cells transfected with control vector (*closed circle, DKD + empty*) with the MTT viability assay. (C) Comparison of the average IC_50_ values for 5-FU between NSUN2 and METTL1 knockdown cells co-expressing NSUN2 and METTL1 and control vector-transfected cells. NS, not significant. *Significant difference compared with control vector-transfected cells (*P*<0.05).

NSUN2 is phosphorylated at Ser139 by Aurora-B, and the phosphorylation of this site is critical for repression of its methyltransferase activity [17]. The same regulation of enzymatic activity has been reported for METTL1, which is phosphorylated at Ser27 by Akt [13]. If decreased sensitivities to 5-FU by double knockdown of NSUN2 and METTL1 are due to decreased methyltransferase activities resulting from decreased expression levels of both proteins, co-expression of enzymatic active forms of both methyltransferases in NSUN2 and METTL1 knockdown cells may confer resistance to 5-FU. To test this hypothesis, we established stable clones originating from UTR-targeting shRNA-mediated NSUN2 and METTL1 knockdown cells with co-expression of both NSUN2 and METTL1 using expression plasmids containing only the wild type coding region of NSUN2 and METTL1 or genetically engineered to encode phosphorylation- or dephosphorylation-mimetic proteins (Figure 6A). Then 5-FU sensitivities were examined in these stable clones. As shown in Figure 6B and 6C, co-expression of wild type proteins NSUN2 and METTL1 conferred protection against 5-FU-induced cytotoxicity on NSUN2 and METTL1 knockdown cells. Compared to wild type, dephosphorylation-mimic forms were more effective (Figure 6C). On the other hand, phosphorylation-mimic forms had no effect (Figure 6B and 6C). Thus, the combined activity of NSUN2 and METTL1 seems to be critical for 5-FU sensitivity of cancer cells, suggesting the presence of a conserved RTD-like pathway regulated by NSUN2 and METTL1 for participating in a surveillance system for tRNA quality control in human.

## Discussion

### NUSN2 is not a critical regulator of cancer cell growth

The upregulated expression of NSUN2 in cancer cells is notable because the overexpression is accompanied by gene copy number gain [14], [15]. NSUN2 has an enzyme activity that can methylate tRNA [17]. Over the past several decades, many reports have shown elevations of tRNA methyltranferase activity and tRNA level in cancer cells [28], [29]. These observations indicate tRNAs as potential biomarkers for tumor progression and malignancy. Yet, there have been no mechanistic explanation linking increased expression of modifier enzymes of tRNAs to growth advantage of cancer cells. In this study we revealed that NSUN2 is not a critical regulator of cancer cell growth. We conclusively show that forced overexpression and knockdown of NSUN2 do not impact proliferation and cancerous growth properties in soft agar. Indeed, forced overexpression of NSUN2 did not have any oncogenic activity per se and did not potentiate *ras*-induced *in vitro* neoplastic transformation in BALB/c 3T3 (Figure S6).

Although NSUN2 overexpression does not act as oncogene, the likelihood that NSUN2 overexpression might confer a growth advantage or a cancer stem cell property in cancer cells remains. Through the generation and phenotypic analysis of knockout mice, murine NSUN2 as a target of proto-oncogene Myc [26] is required to stem cell growth and normal stem cell differentiation in skin and is also required to testis differentiation [27]. NSUN2 catalyzes the formation of cytosine-5-methylation in nucleic acids including tRNA, rRNA, coding and non-coding RNA, and possibly genome DNA [17], [30]–[34]. The altered expression levels of NUSUN2 might affect cell properties of certain tissues in an epigenetic fashion. Notably, mutations in *NSUN2* gene resulting in the gene expression defect are found in patients with intelligence deficiencies [35]–[37].

Previous studies suggest an alternative mechanism by which NSUN2 overexpression contributes to cancer development. NSUN2 is phosphorylated by Aurora-B, resulting in the repression of enzymatic activity, and distributed to the cytoplasm for participating in spindle stability during mitotic cells [17], [38]. Although the elucidation of the roles of NSUN2 overexpression in cancer cells is still challenging, mitotic spindle-related NSUN2 functions involved in chromosome segregation processes might induce chromosome instability leading to cancer progression.

### Cooperation between NSUN2 and METTL1 is responsible for determining 5-FU sensitivity in cancer cells

In yeast *trm4*/*trm8* double mutant, certain hypo-modified tRNAs, particularly tRNA(Val^AAC^), are destabilized by RTD pathway [21]. Due to this tRNA quality check, the double mutant is temperature-sensitive. The RTD pathway in yeast can also be triggered without coincident temperature sensitivity or without loss of modifications in different tRNA variants [23]. In our present study, double knockdown of NSUN2 and METTL1 in HeLa cells did not affect heat stress-induced cytotoxicity. Thus, it is likely that RTD is not evolutionarily conserved with respect to heat stress-induced rapid tRNA decay involving Trm4 and Trm8.

Wild type yeast cells are temperature-sensitive in the presence of higher 5-FU concentrations. Moreover, synthetic interaction between *trm8* and *pus1* (pseudouridylation defective) mutations is observed under heat stress [25]. Reduced pseudouridylation is the most likely cause of 5-FU-induced tRNA damage(s) that mediate tRNA destabilization [25]. Similarly, HeLa cells is 5-FU-sensitive under heat stress. However, the 5-FU-sensitive phenotype of yeast *trm8* mutant under heat stress is not evolutionarily conserved because heat stress potentiates the sensitivity of those cells to the cytotoxicity of 5-FU in the same way as control cells (Figure S7).

**Figure 7:**
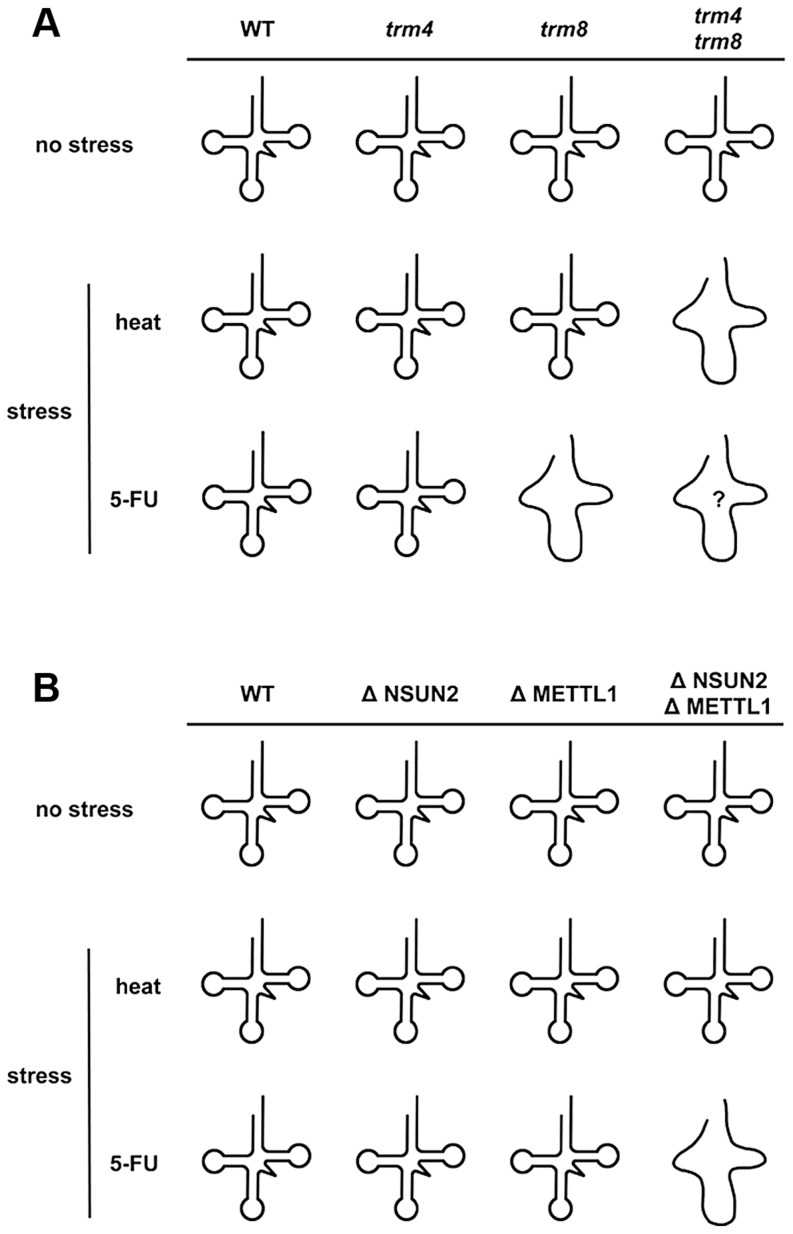
Model for how loss of NSUN2- and METTL1-dependent tRNA(Val^AAC^) modifications causes 5-FU-induced tRNA destabilization. (A) Synthetic interaction between *trm4* and *trm8* mutations in yeast. The temperature-sensitive phenotype of yeast *trm4*/*trm8* double mutant is due to rapid decay of hypomodified tRNA. A melted loop indicates destabilized tRNA. Although 5-FU-induced tRNA damage(s) that mediate tRNA destabilization remains to be fully determined, reduced pseudouridylation is the most likely cause of this. Only *trm8* mutant, but not *trm4* mutant, is sensitive to 5-FU. And so hypomodified tRNA caused by *trm4* and *trm8* mutations is probably hypersensitive to 5-FU (shown as a melted loop with question mark). (B) Cooperative effect between NSUN2 and METTL1 in HeLa cells. Hypomodified tRNA caused by double knockdown of NSUN2 and METTL1 is not heat-sensitive but is 5-FU-sensitive. A melted loop indicates destabilized tRNA.

At normal temperature in the presence of 5-FU, analysis of double mutants showed that yeast *trm8* genetically interacts with other genes encoding tRNA modification activities such as *trm10*, *pus1*, and *mod5* [25]. Only *trm8* mutant, but not *trm4* mutant, is found to be 5-FU sensitive [25]. Although it is not clear whether yeast *trm4*/*trm8* double mutant displays hypersensitivity to 5-FU, tRNA modifying enzymes should be important factors for determining 5-FU sensitivity in yeast. Here, our experiments using HeLa cells demonstrated that these RTD-related tRNA modifying enzymes are associated with 5-FU sensitivity. Apparently the effect of 5-FU is different in yeast when compared to HeLa cells. Knockdown of NSUN2 and METTL1 in HeLa cells potentiates sensitivity of the cells to 5-FU, whereas heat stress of cells revealed no effects. In contrast, yeast tRNA modification mutants show similar synthetic interactions for temperature sensitivity and sensitivity to 5-FU. These differences are related to tRNA stability (Figure 5). This result supports the idea that 5-FU-substituted and hypomodified tRNA that is caused by knockdown of tRNA methylases (NSUN2 and METTL1) after 5-FU exposure is possibly monitored and checked by RTD pathway in mammalian cells. Although the sites of specific tRNA modifications have not been yet determined in HeLa cells used here, METTL1 and NSUN2 target sites are mostly conserved between mammals and yeasts [32], [39]. In our model (Figure 7), loss of tRNA modification causes tRNA destabilization, which is detected as temperature sensitivity in yeast but not in mammals, but may be detected as 5-FU sensitivity in mammals.

Interestingly, in our study, degradation of tRNA(iMet) is heat-sensitive but is also 5-FU-sensitive (Figure 5). From these data, the tRNA(iMet) stability is not likely to be related to tRNA modifications generated by NSUN2 and METTL1. This observation is of particular interest when considering stress responses that undergo a quality control check by RTD, because tRNA(iMet) stability is not affected by stresses other than heat or 5-FU, such as ethanol, hydrogen peroxide (oxidative stresses), low-pH (pH 5.0), cycloheximide, and sodium chloride (high salt concentrations) [40].

Loss of tRNA(iMet) at high temperature was reported previously by Watanabe et al. who has shown that degradation of initiator tRNA, tRNA(iMet), depends on Xrn1/2 nucleases [40]. Yeast RTD pathway that includes Rat1 and Xrn2, monitors the structural integrity of the acceptor and T-stem of tRNA and degrades unstable tRNA species. Since the problem of the structural stabilization of the acceptor and T-stem in tRNA(iMet) in HeLa has been not clarified, Watanabe and colleagues suggest that the degradation pathway in human may resemble the RTD pathway in yeast. A role of Xrn1/2 in degradation of tRNA(Val^AAC^) in HeLa exposed to 5-FU is unknown. Yeast Trm4 and Trm8 are required for protecting yeast tRNA against RTD, but the functional analogy of their mammalian orthologs NSUN2 and METTL1 would need further study to determine more precisely.

DNA and RNA species other than tRNA, such as rRNA, snRNA, mRNA and pre-mRNA, are targets for 5-FU [1]. Those are also targets for NSUN2 with broad substrate specificity [17], [26], [31]–[34]. However, the possibility that the nucleic acid species other than tRNA are involved in the NSUN2 and METTL1-dependent potentiation mechanism under 5-FU treatment is probably eliminated, because METTL1 is evolutionarily conserved and catalyzes the formation of N7-methylguanine at position 46 in tRNA in a substrate-specific manner [13], [20], [24].

### tRNA and cancer

It has long been suggested that deregulation of translation contributes to cancer development [41], [42]. Forced overexpression of components of translation machinery can lead to cell transformation [43], [44]. Elevations of tRNA methyltransferase activities and elevated expression levels of tRNA have also long been recognized in cancer cells [28], [29], [45], [46]. Although such several circumstantial evidences suggests that deregulation of translation might be a common cause for human cancer, one viewpoint still argues that such tRNA deregulation might be only characteristics of cancer cells like a by-product, which is associated with increased proliferation and elevated levels of protein synthesis. The tRNA overexpression that leads to increase the translation efficiency of genes is indeed characteristic of cancer cells but does not necessarily need to be related to their growth advantage. In our view, based on the present study, tRNA modifications are likely to contribute to cancer cell survival under certain stresses that interferes with tRNA stability. We therefore envisage new approaches implementing RNA-methyltransferase inhibitors or tRNA-modifying molecules as amplifier for chemotherapy of cancer.

## Materials and Methods

### Cells, cell culture and reagents

The human cervical cancer cell line HeLa was provided by the late Professor Masakatsu Horikawa, Faculty of Pharmaceutical Sciences, Kanazawa University [47]. The cells were cultured in Dulbecco's modified Eagle's minimum essential medium containing 10% fetal bovine serum at 37°C in a humidified atmosphere of 5% CO_2_ and 95% air.

5-FU (Nakarai Tesque, Kyoto, Japan) was dissolved in distilled water. Cisplatin (Sigma-Aldrich, St. Louis, MO, USA) was dissolved in phosphate buffered saline solution without Ca^2+^ and Mg^2+^. Paclitaxel (Sigma-Aldrich, St. Louis, MO, USA) was dissolved in dimethyl sulfoxide. The chemical treatment was performed carefully under a protocol approved for evaluation in *in vitro* cultured cells [48].

### Antibodies

Rabbit polyclonal anti-NSUN2 [17] and anti-METTL1 (ProteinTech Group, Chicago, IL, USA) antibodies were used to detect endogenous tRNA modifying enzymes. Monoclonal anti-Xpress (Invitrogen, Carlsbad, CA, USA) and anti-α-tubulin (Cedarlane Laboratories, Burlington, Ontario, Canada) antibodies were also used.

### Protein extraction and immunoblot analysis

Whole cell lysates were prepared using Laemmli SDS-sample buffer. Protein concentration of lysed cells was quantified via the Bradford assay (Bio-Rad Protein Assay, Bio-Rad Laboratories, Hercules, CA, USA). Proteins were resolved by sodium dodecyl sulfate-polyacrylamide gel electrophoresis (20 µg/lane), electroblotted onto a polyvinylidene difluoride membrane, Immobilon P (Millipore Corporation, Billerica, MA, USA), using an electroblotter (ATTO, Tokyo, Japan), and incubated with an appropriate antibody. The immunoblots were developed using electrogenerated chemiluminescence reagents kit (GE-Amersham, Princeton, NJ, USA).

### Plasmids

Expression plasmids for Xpress-tagged NSUN2 and its constitutively dephosphorylated and phosphorylated forms (Xpress-tagged NSUN2-S139A and Xpress-tagged NSUN2-S139E) have been described previously [17]. The coding region of METTL1 cDNA (GenBank: BC000550.1, Open Biosystems, Huntsville, AL, USA) was amplified by polymerase chain reaction and subcloned into pcDNA3.1/His, generating plasmid pcDNA3.1-Xpress-METTL1. The following mutant METTL1 expression plasmids were also constructed: pcDNA3.1-Xpress-METTL1-SA, producing dephosphorylation-mimic METTL1-S27A, in which Ser27 of METTL1 was replaced with Ala by site-directed mutation at T79G; and pcDNA3.1-Xpress-METTL1-SE, producing phosphorylation-mimic METTL1-S27E in which Ser27 of METTL1 was replaced with Glu by site-directed mutation at T79G, C80A and C81A.

### Stable transfectants with forced overexpression of tRNA modifying enzymes

HeLa cells were transfected with constructs encoding tRNA modifying enzymes or control vector by using lipofectamine 2000 (Invitrogen, Carlsbad, CA, USA). G418-resistant colonies (over twenty colonies) were cloned in each transfection, and the protein expression of each clone was checked by immunoblot analysis. Five independent clones that overexpressed Xpress-tagged proteins were pooled and used as a stable transfectant.

### Knockdown of endogeneous tRNA modifying enzymes using short hairpin RNA (shRNA)

Oligonucleotides targeted to the 5′-untranslated region (UTR) of NSUN2 and METTL1 were synthesized, and were ligated into pSUPERIOR.PURO (OligoEngine, Seattle, WA, USA). Puromycin-resistant colonies (thirty colonies) were cloned in each transfection, and the protein expression of each clone was checked by immunoblot analysis as shown previously [17]. Five independent clones that repress expression of endogenous tRNA modifying enzymes were pooled and used as a knockdown clone.

### Analysis of cell growth curves

Doubling times were calculated from growth curves in logarithmic growth phase.

### Ability to form colonies in semisolid medium

Colony-forming abilities in semisolid medium were determined by the method described previously [49]. Anchorage-independent colonies grown in 0.2% washed agar medium were counted.

### Analysis of cell viability with water-soluble tetrazolium-1 (WST-1) assay

Cells were inoculated into 96-well plates. After 4 h of culture, each drug solution was added. For hyperthermia experiments, cells were cultured at 43°C for various periods. The treated cells were cultured for additional 72 h. Cell viability was determined by WST-1 assay (Roche Applied Science, Indianapolis, IN, USA). The IC_50_ value represents the drug concentration resulting in 50% viability.

### Analysis of cell viability with colony formation assay

Colony formation assay [50], also known as clonogenic assay, was used to confirm the cytotoxic effect of 5-FU. Cells were plated onto a plastic surface and allowed to attach for 24 h. Each drug solution was added directly into each dish, as mentioned previously[48]. Colonies formed were stained and counted. The IC_50_ value represents the drug concentration resulting in 50% survival.

### Northern blot analysis of RNA

Total RNA was isolated from HeLa cells treated with heat stress or 5-FU by using RNAiso Plus (Takara-Clontech, Tokyo, Japan). RNA gel electrophoresis and Northern blot analysis identified tRNA(Val^AAC^), tRNA(iMet), tRNA(eMet), and 5S rRNA by using each specific labeled probes as described below. Quantification of band intensity was calculated by Multi Gauge (Fujifilm, Tokyo, Japan). The probe sequences are tRNA(Val^AAC^): GGACCTTTCGCGTGTTAGG, tRNA(iMet): GCAGAGGATGGTTTCGATCCATC, tRNA(eMet): CCCCGTGTGAGGATCGAACTCAC, and 5S rRNA: CAGGGTGGTATGGCCGTAGAC.

### Statistics

All experiments to obtain quantitative data were repeated independently three times (n = 3). Differences between values were analyzed using a two-tailed Welch's *t*-test. *P*-values of <0.05 were considered significant. Data is presented as the mean +/− one standard deviation (SD).

## Supporting Information

Figure S1 Effects of decreased NSUN2 expression on cell growth and on anchorage-independent growth. HeLa cells and their derived five clones transfected with NSUN2-shRNA #1, #2, or #3 were used. Expression levels of NSUN2 were previously checked by immunoblot analysis (see Figure S4 in ref. [17]). NSUN2 expression is completely repressed in clones #2-1, #2-8, and #3-5, moderately repressed in clone #1-7, and not repressed in clone #3-3. (A) *In vitro* growth curves of these clones (#1-7, #2-1, #2-8 #3-3, and #3-5) and parental HeLa cells. (B) Colony-forming abilities of these clones (lanes *#1-7, #2-1, #2-8 #3-3, and #3-5*) and parental HeLa cells (lane *HeLa*) in 0.2% washed agar medium. All experiments to obtain quantitative data were repeated independently three times (n = 3). Data throughout this study represent the mean ± SD for three independent experiments, and error bars represent the SD. Difference between values were analyzed using a two-tailed Welch's *t*-test. *P*-values of <0.05 were considered significant. NS, not significant.
(TIF)

Figure S2 Effects of increased NSUN2 expression on cell growth and on anchorage-independent growth. (A) Immunoblot analysis of proteins from Xpress-NSUN2-overexpressing cells (lane *Xpress-NSUN2*) and control vector-transfected cells (lane *empty*) with anti-Xpress, anti-NSUN2 and anti-α-tubulin antibodies. Five independent clones that overexpressed Xpress-NSUN2 or were transfected with the empty vector were pooled and used as a stable transfectant. (B) *In vitro* doubling times of Xpress-NSUN2-overexpressing cells (lane *Xpress-NSUN2*) and control vector-transfected cells (lane *empty*). (C) Colony-forming abilities of Xpress-NSUN2-overexpressing cells (lane *Xpress-NSUN2*) and control vector-transfected cells (lane *empty*) in 0.2% washed agar medium. NS, not significant.
(TIF)

Figure S3 Effects of decreased NSUN2 expression on cell growth and on anchorage-independent growth. Newly isolated clones transfected with NSUN2-shRNA targeted to UTR were used. (A) Immunoblot analysis of proteins from NSUN2 knockdown cells (lane *NSUN2*) and scrambled control vector-transfected cells (lane *scrambled*) with anti-NSUN2 and anti-α-tubulin antibodies. Five independent clones that decreased endogenous NSUN2 expression or were transfected with the scrambled control vector were pooled and used as a stable transfectant. (B) *In vitro* doubling times of NSUN2 knockdown cells (lane *NSUN2*) and scrambled control vector-transfected cells (lane *scrambled*). (C) Colony-forming abilities of NSUN2 knockdown cells (lane *NSUN2*) and scrambled control vector-transfected cells (lane *scrambled*) in 0.2% washed agar medium. NS, not significant.
(TIF)

Figure S4 Effects of increased NSUN2 and METTL1 co-expression on cell growth and on anchorage-independent growth. (A) *In vitro* doubling times of Xpress-METTL1-overexpressing cells (lane *Xpress-METTL1*), Xpress-NSUN2- and Xpress-METTL1-co-overexpressing cells (lane *Xpress-NSUN2 + Xpress-METTL1*) and control vector-transfected cells (lane *empty*). (B) Colony-forming abilities of Xpress-METTL1-overexpressing cells (lane *Xpress-METTL1*), Xpress-NSUN2- and Xpress-METTL1-co-overexpressing cells (lane *Xpress-NSUN2 + Xpress-METTL1*) and control vector-transfected cells (lane *empty*) in 0.2% washed agar medium. NS, not significant.
(TIF)

Figure S5 Effects of decreased expression of NSUN2 and METTL1 on cell growth and on anchorage-independent growth in UTR-targeting shRNA-mediated newly established knockdown cells. (A) *In vitro* doubling times of NSUN2 knockdown cells (lane *NSUN2*), METTL1 knockdown cells (lane *METTL1*), NSUN2 and METTL1 knockdown cells (lane *NSUN2 + METTL1*) and scrambled control vector-transfected cells (lane *scrambled*). (B) Colony-forming abilities of NSUN2 knockdown cells (lane *NSUN2*), METTL1 knockdown cells (lane *METTL1*), NSUN2 and METTL1 knockdown cells (lane *NSUN2 + METTL1*) and scrambled control vector-transfected cells (lane *scrambled*) in 0.2% washed agar medium. NS, not significant.
(TIF)

Figure S6 Effects of overexpression of NSUN2, NSUN2^S139A^, and NSUN2^S139E^ on H-Ras^G12V^-induced cell transformation in vitro. A BALB/c 3T3 A31-1-1 cell transformation assay system was utilized, and quantification of the number of transformed foci was determined using standard criteria [51], [52]. The inset shows an immunoblot of cells transfected with empty vectors (lane *1*), NSUN2 (lane *2*), NSUN2^S139A^ (lane *3*), NSUN2^S139E^ (lane *4*), H-Ras^G12V^ (lane *5*), NSUN2 + H-Ras^G12V^ (lane *6*), NSUN2^S139A^ + H-Ras^G12V^ (lane *7*), and NSUN2^S139E^ + H-Ras^G12V^ (lane *8*). NS, not significant.
(TIF)

Figure S7 Cell survival in response to 5-FU (IC_50_ concentrations) under heat stress (43°C for 1.5 h) in NSUN2 and METTL1 knockdown cells (lane *NSUN2 + METTL1*), and control vector-transfected cells (lane *scrambled*) with the MTT viability assay. NS, not significant.
(TIF)

## References

[1] Longley DB, Harkin DP, Johnston PG (2003) 5-fluorouracil: mechanisms of action and clinical strategies. Nat Rev Cancer 3: 330–338. 12724731 10.1038/nrc1074

[2] Benz C, Tillis T, Tattelman E, Cadman E (1982) Optimal schedule of methotrexate and 5-fluorouracil in human breast cancer. Cancer Res 42: 2081–2086. 6175406

[3] Bertino JR, Mini E, Fernandes DJ (1983) Sequential methotrexate and 5-fluorouracil: mechanisms of synergy. Semin Oncol 10: 2–5. 6867754

[4] Bonnefoi H, Smith IE, O'Brien ME, Seymour MT, Powles TJ, et al (1996) Phase II study of continuous infusional 5-fluorouracil with epirubicin and carboplatin (instead of cisplatin) in patients with metastatic/locally advanced breast cancer (infusional ECarboF): a very active and well-tolerated outpatient regimen. Br J Cancer 73: 391–396. 8562348 10.1038/bjc.1996.67 PMC2074421

[5] Greco FA, Figlin R, York M, Einhorn L, Schilsky R, et al (1996) Phase III randomized study to compare interferon alfa-2a in combination with fluorouracil versus fluorouracil alone in patients with advanced colorectal cancer. J Clin Oncol 14: 2674–2681. 8874326 10.1200/JCO.1996.14.10.2674

[6] Jones DV Jr, Winn RJ, Brown BW, Levy LB, Pugh RP, et al (1995) Randomized phase III study of 5-fluorouracil plus high dose folinic acid versus 5-fluorouracil plus folinic acid plus methyl-lomustine for patients with advanced colorectal cancer. Cancer 76: 1709–1714. 8625038 10.1002/1097-0142(19951115)76:10<1709::aid-cncr2820761006>3.0.co;2-5

[7] Marsoni S (1995) Fluorouracil and folinic acid in colon cancer. IMPACT Investigators. Lancet 345: 1582–1583. 7791476

[8] Nadal JC, Van Groeningen CJ, Pinedo HM, Peters GJ (1988) In vivo potentiation of 5-fluorouracil by leucovorin in murine colon carcinoma. Biomed Pharmacother 42: 387–393. 3064823

[9] Wolmark N, Bryant J, Smith R, Grem J, Allegra C, et al (1998) Adjuvant 5-fluorouracil and leucovorin with or without interferon alfa-2a in colon carcinoma: National Surgical Adjuvant Breast and Bowel Project protocol C-05. J Natl Cancer Inst 90: 1810–1816. 9839521 10.1093/jnci/90.23.1810

[10] Hung SW, Mody HR, Govindarajan R (2012) Overcoming nucleoside analog chemoresistance of pancreatic cancer: a therapeutic challenge. Cancer Lett 320: 138–149. 22425961 10.1016/j.canlet.2012.03.007 PMC3569094

[11] Shi S, Yao W, Xu J, Long J, Liu C, et al (2012) Combinational therapy: new hope for pancreatic cancer? Cancer Lett 317: 127–135. 22138436 10.1016/j.canlet.2011.11.029

[12] de la Cruz-Morcillo MA, Valero ML, Callejas-Valera JL, Arias-Gonzalez L, Melgar-Rojas P, et al (2012) P38MAPK is a major determinant of the balance between apoptosis and autophagy triggered by 5-fluorouracil: implication in resistance. Oncogene 31: 1073–1085. 21841826 10.1038/onc.2011.321

[13] Cartlidge RA, Knebel A, Peggie M, Alexandrov A, Phizicky EM, et al (2005) The tRNA methylase METTL1 is phosphorylated and inactivated by PKB and RSK in vitro and in cells. EMBO J 24: 1696–1705. 15861136 10.1038/sj.emboj.7600648 PMC1142581

[14] Frye M, Dragoni I, Chin SF, Spiteri I, Kurowski A, et al (2010) Genomic gain of 5p15 leads to over-expression of Misu (NSUN2) in breast cancer. Cancer Lett 289: 71–80. 19740597 10.1016/j.canlet.2009.08.004

[15] Okamoto M, Hirata S, Sato S, Koga S, Fujii M, et al (2012) Frequent increased gene copy number and high protein expression of tRNA (cytosine-5-)-methyltransferase (NSUN2) in human cancers. DNA Cell Biol 31: 660–671. 22136356 10.1089/dna.2011.1446

[16] Wikman H, Nymark P, Vayrynen A, Jarmalaite S, Kallioniemi A, et al (2005) CDK4 is a probable target gene in a novel amplicon at 12q13.3-q14.1 in lung cancer. Genes Chromosomes Cancer 42: 193–199. 15543620 10.1002/gcc.20122

[17] Sakita-Suto S, Kanda A, Suzuki F, Sato S, Takata T, et al (2007) Aurora-B regulates RNA methyltransferase NSUN2. Mol Biol Cell 18: 1107–1117. 17215513 10.1091/mbc.E06-11-1021 PMC1805108

[18] Motorin Y, Grosjean H (1999) Multisite-specific tRNA:m5C-methyltransferase (Trm4) in yeast Saccharomyces cerevisiae: identification of the gene and substrate specificity of the enzyme. RNA 5: 1105–1118. 10445884 10.1017/s1355838299982201 PMC1369833

[19] Wu P, Brockenbrough JS, Paddy MR, Aris JP (1998) NCL1, a novel gene for a non-essential nuclear protein in Saccharomyces cerevisiae. Gene 220: 109–117. 9767141 10.1016/s0378-1119(98)00330-8

[20] Alexandrov A, Martzen MR, Phizicky EM (2002) Two proteins that form a complex are required for 7-methylguanosine modification of yeast tRNA. RNA 8: 1253–1266. 12403464 10.1017/s1355838202024019 PMC1370335

[21] Alexandrov A, Chernyakov I, Gu W, Hiley SL, Hughes TR, et al (2006) Rapid tRNA decay can result from lack of nonessential modifications. Mol Cell 21: 87–96. 16387656 10.1016/j.molcel.2005.10.036

[22] Chernyakov I, Whipple JM, Kotelawala L, Grayhack EJ, Phizicky EM (2008) Degradation of several hypomodified mature tRNA species in Saccharomyces cerevisiae is mediated by Met22 and the 5′-3′ exonucleases Rat1 and Xrn1. Genes Dev 22: 1369–1380. 18443146 10.1101/gad.1654308 PMC2377191

[23] Whipple JM, Lane EA, Chernyakov I, D'Silva S, Phizicky EM (2011) The yeast rapid tRNA decay pathway primarily monitors the structural integrity of the acceptor and T-stems of mature tRNA. Genes Dev 25: 1173–1184. 21632824 10.1101/gad.2050711 PMC3110955

[24] Bahr A, Hankeln T, Fiedler T, Hegemann J, Schmidt ER (1999) Molecular analysis of METTL1, a novel human methyltransferase-like gene with a high degree of phylogenetic conservation. Genomics 57: 424–428. 10329009 10.1006/geno.1999.5780

[25] Gustavsson M, Ronne H (2008) Evidence that tRNA modifying enzymes are important in vivo targets for 5-fluorouracil in yeast. RNA 14: 666–674. 18314501 10.1261/rna.966208 PMC2271368

[26] Frye M, Watt FM (2006) The RNA methyltransferase Misu (NSun2) mediates Myc-induced proliferation and is upregulated in tumors. Curr Biol 16: 971–981. 16713953 10.1016/j.cub.2006.04.027

[27] Blanco S, Kurowski A, Nichols J, Watt FM, Benitah SA, et al (2011) The RNA-methyltransferase Misu (NSun2) poises epidermal stem cells to differentiate. PLoS Genet 7: e1002403. 22144916 10.1371/journal.pgen.1002403 PMC3228827

[28] Kuchino Y, Borek E (1976) Changes in transfer RNA's in human malignant trophoblastic cells (BeWo line). Cancer Res 36: 2932–2936. 179710

[29] Pavon-Eternod M, Gomes S, Geslain R, Dai Q, Rosner MR, et al (2009) tRNA over-expression in breast cancer and functional consequences. Nucleic Acids Res 37: 7268–7280. 19783824 10.1093/nar/gkp787 PMC2790902

[30] Auxilien S, Guerineau V, Szweykowska-Kulinska Z, Golinelli-Pimpaneau B (2012) The human tRNA m (5) C methyltransferase Misu is multisite-specific. RNA Biol 9: 1331–1338. 22995836 10.4161/rna.22180 PMC3597573

[31] Squires JE, Patel HR, Nousch M, Sibbritt T, Humphreys DT, et al (2012) Widespread occurrence of 5-methylcytosine in human coding and non-coding RNA. Nucleic Acids Res 40: 5023–5033. 22344696 10.1093/nar/gks144 PMC3367185

[32] Khoddami V, Cairns BR (2013) Identification of direct targets and modified bases of RNA cytosine methyltransferases. Nat Biotechnol 31: 458–464. 23604283 10.1038/nbt.2566 PMC3791587

[33] Hussain S, Sajini AA, Blanco S, Dietmann S, Lombard P, et al (2013) NSun2-mediated cytosine-5 methylation of vault noncoding RNA determines its processing into regulatory small RNAs. Cell Rep 4: 255–261. 23871666 10.1016/j.celrep.2013.06.029 PMC3730056

[34] Zhang X, Liu Z, Yi J, Tang H, Xing J, et al (2012) The tRNA methyltransferase NSun2 stabilizes p16INK(4) mRNA by methylating the 3′-untranslated region of p16. Nat Commun 3: 712. 22395603 10.1038/ncomms1692 PMC3509206

[35] Martinez FJ, Lee JH, Lee JE, Blanco S, Nickerson E, et al (2012) Whole exome sequencing identifies a splicing mutation in NSUN2 as a cause of a Dubowitz-like syndrome. J Med Genet 49: 380–385. 22577224 10.1136/jmedgenet-2011-100686 PMC4771841

[36] Khan MA, Rafiq MA, Noor A, Hussain S, Flores JV, et al (2012) Mutation in NSUN2, which encodes an RNA methyltransferase, causes autosomal-recessive intellectual disability. Am J Hum Genet 90: 856–863. 22541562 10.1016/j.ajhg.2012.03.023 PMC3376419

[37] Abbasi-Moheb L, Mertel S, Gonsior M, Nouri-Vahid L, Kahrizi K, et al (2012) Mutations in NSUN2 cause autosomal-recessive intellectual disability. Am J Hum Genet 90: 847–855. 22541559 10.1016/j.ajhg.2012.03.021 PMC3376487

[38] Hussain S, Benavente SB, Nascimento E, Dragoni I, Kurowski A, et al (2009) The nucleolar RNA methyltransferase Misu (NSun2) is required for mitotic spindle stability. J Cell Biol 186: 27–40. 19596847 10.1083/jcb.200810180 PMC2712989

[39] Towns WL, Begley TJ (2012) Transfer RNA methytransferases and their corresponding modifications in budding yeast and humans: activities, predications, and potential roles in human health. DNA Cell Biol 31: 434–454. 22191691 10.1089/dna.2011.1437 PMC3322404

[40] Watanabe K, Miyagawa R, Tomikawa C, Mizuno R, Takahashi A, et al (2013) Degradation of initiator tRNAMet by Xrn1/2 via its accumulation in the nucleus of heat-treated HeLa cells. Nucleic Acids Res 41: 4671–4685. 23471000 10.1093/nar/gkt153 PMC3632136

[41] Ruggero D, Pandolfi PP (2003) Does the ribosome translate cancer? Nat Rev Cancer 3: 179–192. 12612653 10.1038/nrc1015

[42] Pandolfi PP (2004) Aberrant mRNA translation in cancer pathogenesis: an old concept revisited comes finally of age. Oncogene 23: 3134–3137. 15094762 10.1038/sj.onc.1207618

[43] Lazaris-Karatzas A, Montine KS, Sonenberg N (1990) Malignant transformation by a eukaryotic initiation factor subunit that binds to mRNA 5′ cap. Nature 345: 544–547. 2348862 10.1038/345544a0

[44] Tatsuka M, Mitsui H, Wada M, Nagata A, Nojima H, et al (1992) Elongation factor-1 alpha gene determines susceptibility to transformation. Nature 359: 333–336. 1383827 10.1038/359333a0

[45] Borek E, Kerr SJ (1972) Atypical transfer RNA's and their origin in neoplastic cells. Adv Cancer Res 15: 163–190. 4553230 10.1016/s0065-230x(08)60374-7

[46] Pavon-Eternod M, Gomes S, Rosner MR, Pan T (2013) Overexpression of initiator methionine tRNA leads to global reprogramming of tRNA expression and increased proliferation in human epithelial cells. RNA 19: 461–466. 23431330 10.1261/rna.037507.112 PMC3677255

[47] Suzuki F, Horikawa M (1973) A replica plating method of cultured mammalian cells. Methods Cell Biol 6: 127–142. 4585078 10.1016/s0091-679x(08)60050-3

[48] Kakunaga T (1981) Approaches toward developing a human transformation assay system. In: Myron AM, Nawin CM, editors. Mammalian Cell Transformation by Chemical Carcinogens. Washington, DC, U.S.A.: Senate Press. pp. 355–382.

[49] Suzuki F, Suzuki K, Nikaido O (1984) An improved soft agar method for determining neoplastic transformation in vitro. J Tissue Cult Methods 8: 109–113.

[50] Franken NA, Rodermond HM, Stap J, Haveman J, van Bree C (2006) Clonogenic assay of cells in vitro. Nat Protoc 1: 2315–2319. 17406473 10.1038/nprot.2006.339

[51] Tatsuka M, Sato S, Kitajima S, Suto S, Kawai H, et al (2005) Overexpression of Aurora-A potentiates HRAS-mediated oncogenic transformation and is implicated in oral carcinogenesis. Oncogene 24: 1122–1127. 15592510 10.1038/sj.onc.1208293

[52] Kanda A, Kawai H, Suto S, Kitajima S, Sato S, et al (2005) Aurora-B/AIM-1 kinase activity is involved in Ras-mediated cell transformation. Oncogene 24: 7266–7272. 16027732 10.1038/sj.onc.1208884

