# Supplementary figures and images for: tRNA Modifying Enzymes, NSUN2 and METTL1, Determine Sensitivity to 5-Fluorouracil in HeLa Cells
Source: PLoS Genet. 2014 Sep 18;10(9):e1004639. doi: 10.1371/journal.pgen.1004639 (PMC4169382; doi:10.1371/journal.pgen.1004639)

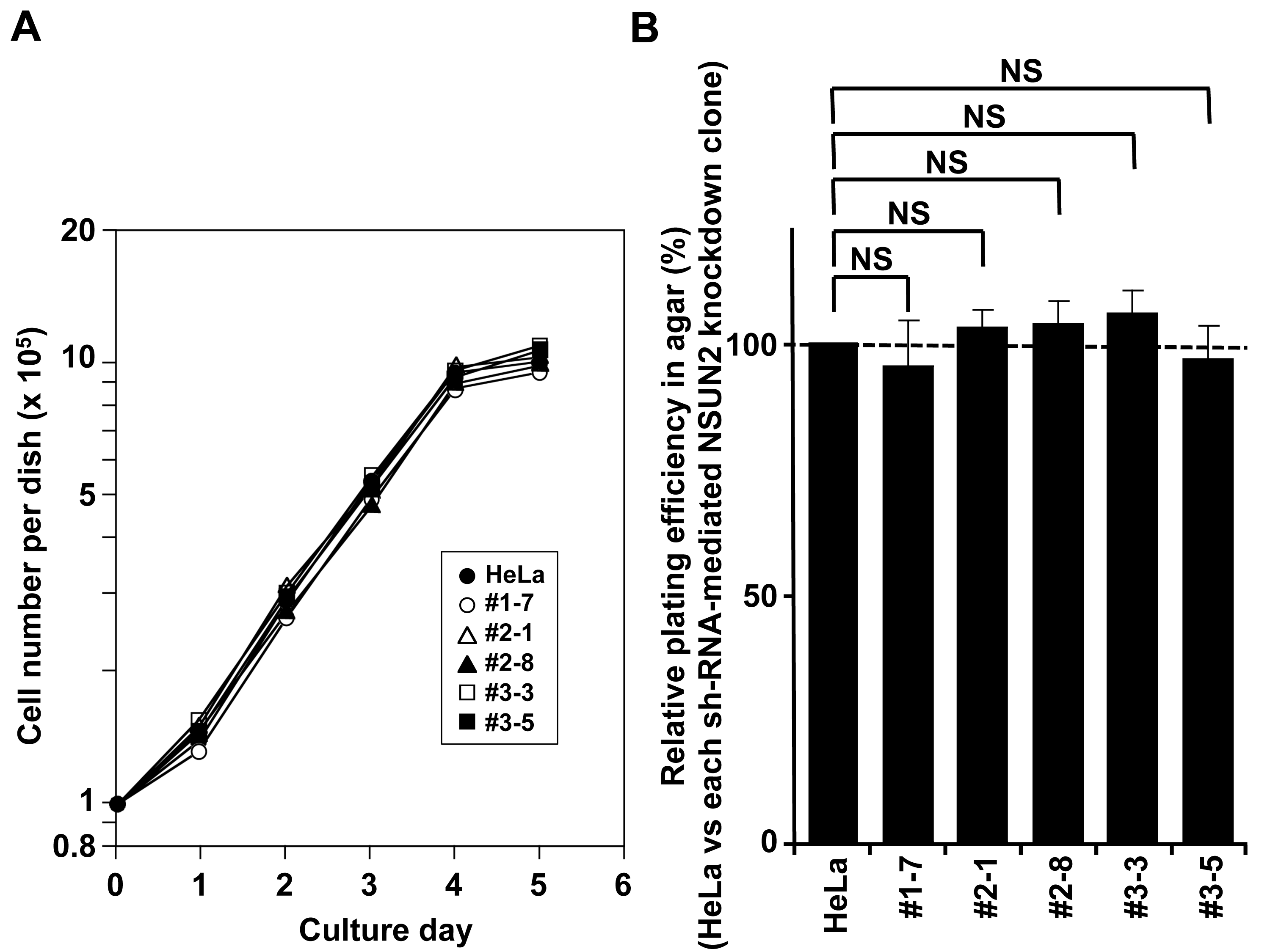

Supplement: Figure S1 — Effects of decreased NSUN2 expression on cell growth and on anchorage-independent growth. HeLa cells and their derived five clones transfected with NSUN2-shRNA #1, #2, or #3 were used. Expression levels of NSUN2 were previously checked by immunoblot analysis (see Figure S4 in ref. [17]). NSUN2 expression is completely repressed in clones #2-1, #2-8, and #3-5, moderately repressed in clone #1-7, and not repressed in clone #3-3. (A) In vitro growth curves of these clones (#1-7, #2-1, #2-8 #3-3, and #3-5) and parental HeLa cells. (B) Colony-forming abilities of these clones (lanes #1-7, #2-1, #2-8 #3-3, and #3-5) and parental HeLa cells (lane HeLa) in 0.2% washed agar medium. All experiments to obtain quantitative data were repeated independently three times (n = 3). Data throughout this study represent the mean ± SD for three independent experiments, and error bars represent the SD. Difference between values were analyzed using a two-tailed Welch's t-test. P-values of <0.05 were considered significant. NS, not significant. (TIF) [file pgen.1004639.s001.tif]

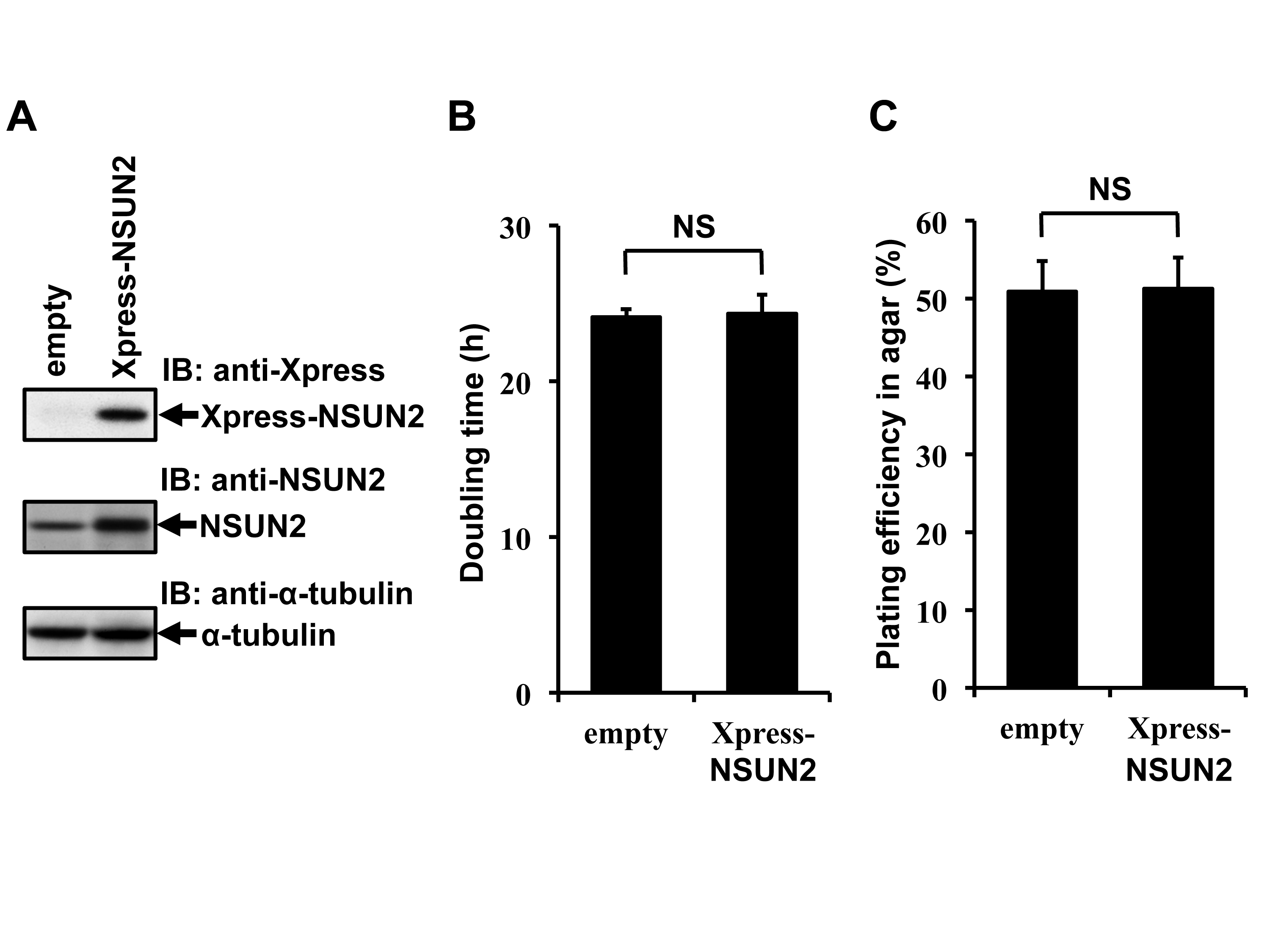

Supplement: Figure S2 — Effects of increased NSUN2 expression on cell growth and on anchorage-independent growth. (A) Immunoblot analysis of proteins from Xpress-NSUN2-overexpressing cells (lane Xpress-NSUN2) and control vector-transfected cells (lane empty) with anti-Xpress, anti-NSUN2 and anti-α-tubulin antibodies. Five independent clones that overexpressed Xpress-NSUN2 or were transfected with the empty vector were pooled and used as a stable transfectant. (B) In vitro doubling times of Xpress-NSUN2-overexpressing cells (lane Xpress-NSUN2) and control vector-transfected cells (lane empty). (C) Colony-forming abilities of Xpress-NSUN2-overexpressing cells (lane Xpress-NSUN2) and control vector-transfected cells (lane empty) in 0.2% washed agar medium. NS, not significant. (TIF) [file pgen.1004639.s002.tif]

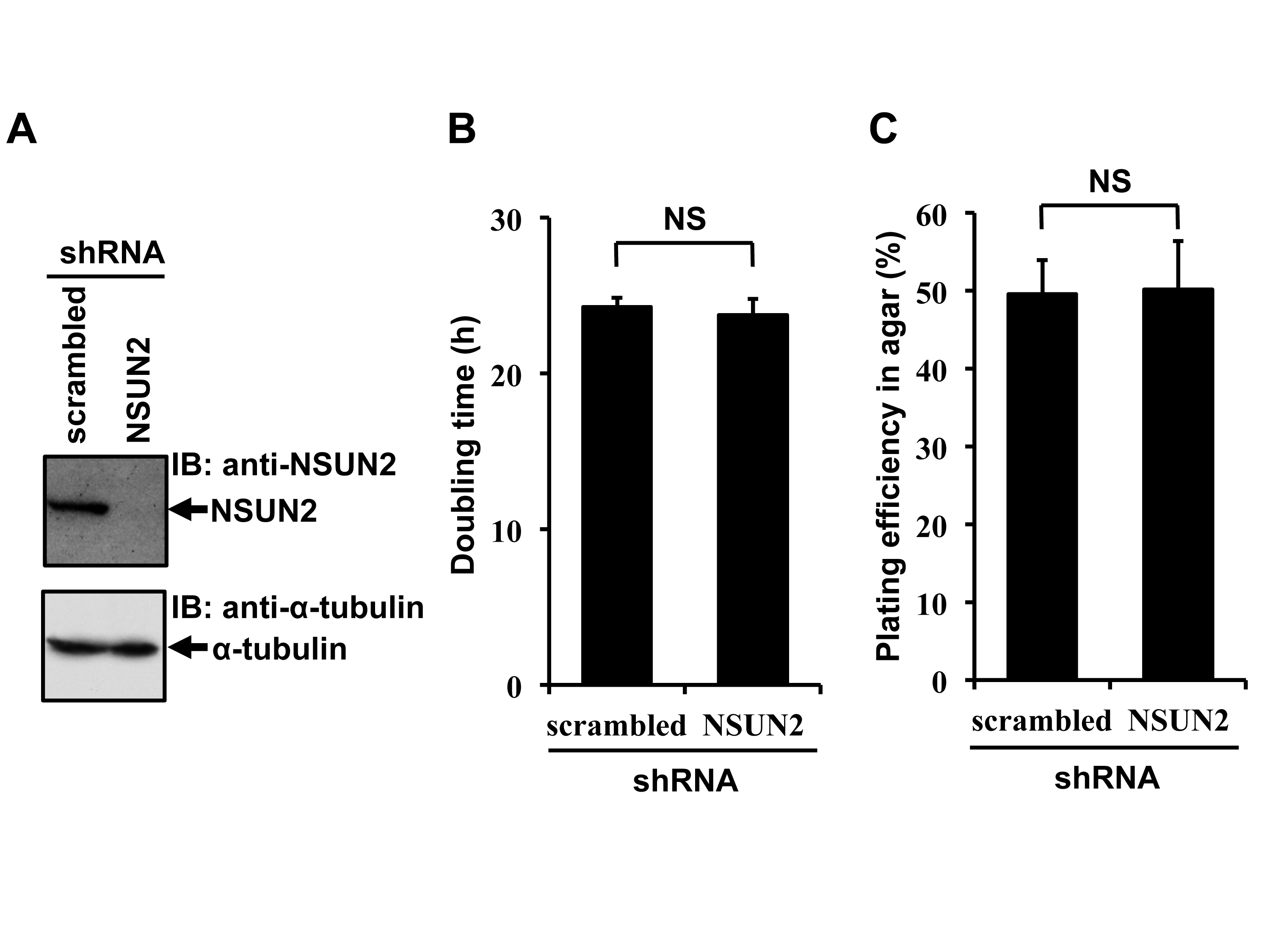

Supplement: Figure S3 — Effects of decreased NSUN2 expression on cell growth and on anchorage-independent growth. Newly isolated clones transfected with NSUN2-shRNA targeted to UTR were used. (A) Immunoblot analysis of proteins from NSUN2 knockdown cells (lane NSUN2) and scrambled control vector-transfected cells (lane scrambled) with anti-NSUN2 and anti-α-tubulin antibodies. Five independent clones that decreased endogenous NSUN2 expression or were transfected with the scrambled control vector were pooled and used as a stable transfectant. (B) In vitro doubling times of NSUN2 knockdown cells (lane NSUN2) and scrambled control vector-transfected cells (lane scrambled). (C) Colony-forming abilities of NSUN2 knockdown cells (lane NSUN2) and scrambled control vector-transfected cells (lane scrambled) in 0.2% washed agar medium. NS, not significant. (TIF) [file pgen.1004639.s003.tif]

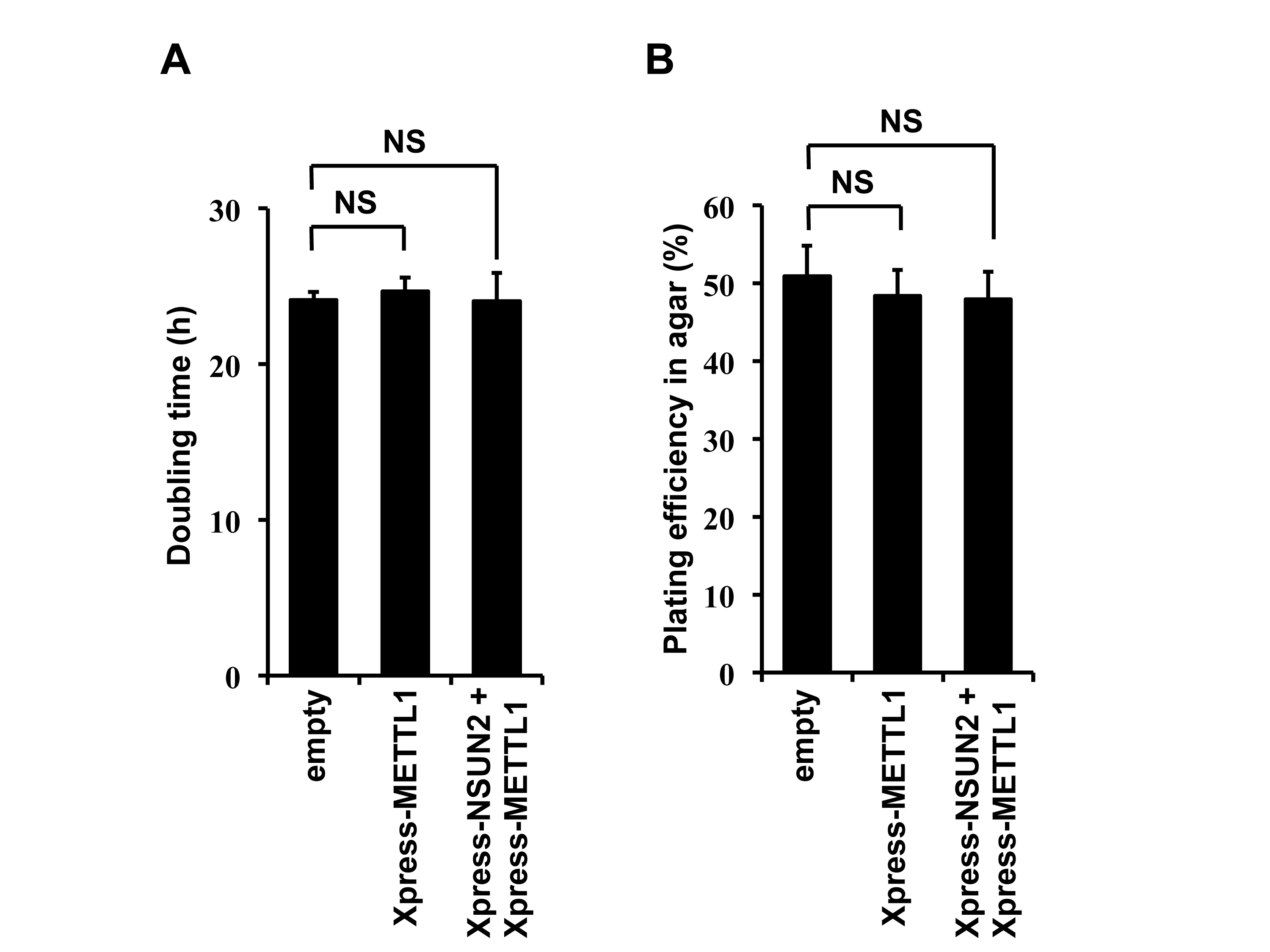

Supplement: Figure S4 — Effects of increased NSUN2 and METTL1 co-expression on cell growth and on anchorage-independent growth. (A) In vitro doubling times of Xpress-METTL1-overexpressing cells (lane Xpress-METTL1), Xpress-NSUN2- and Xpress-METTL1-co-overexpressing cells (lane Xpress-NSUN2 + Xpress-METTL1) and control vector-transfected cells (lane empty). (B) Colony-forming abilities of Xpress-METTL1-overexpressing cells (lane Xpress-METTL1), Xpress-NSUN2- and Xpress-METTL1-co-overexpressing cells (lane Xpress-NSUN2 + Xpress-METTL1) and control vector-transfected cells (lane empty) in 0.2% washed agar medium. NS, not significant. (TIF) [file pgen.1004639.s004.tif]

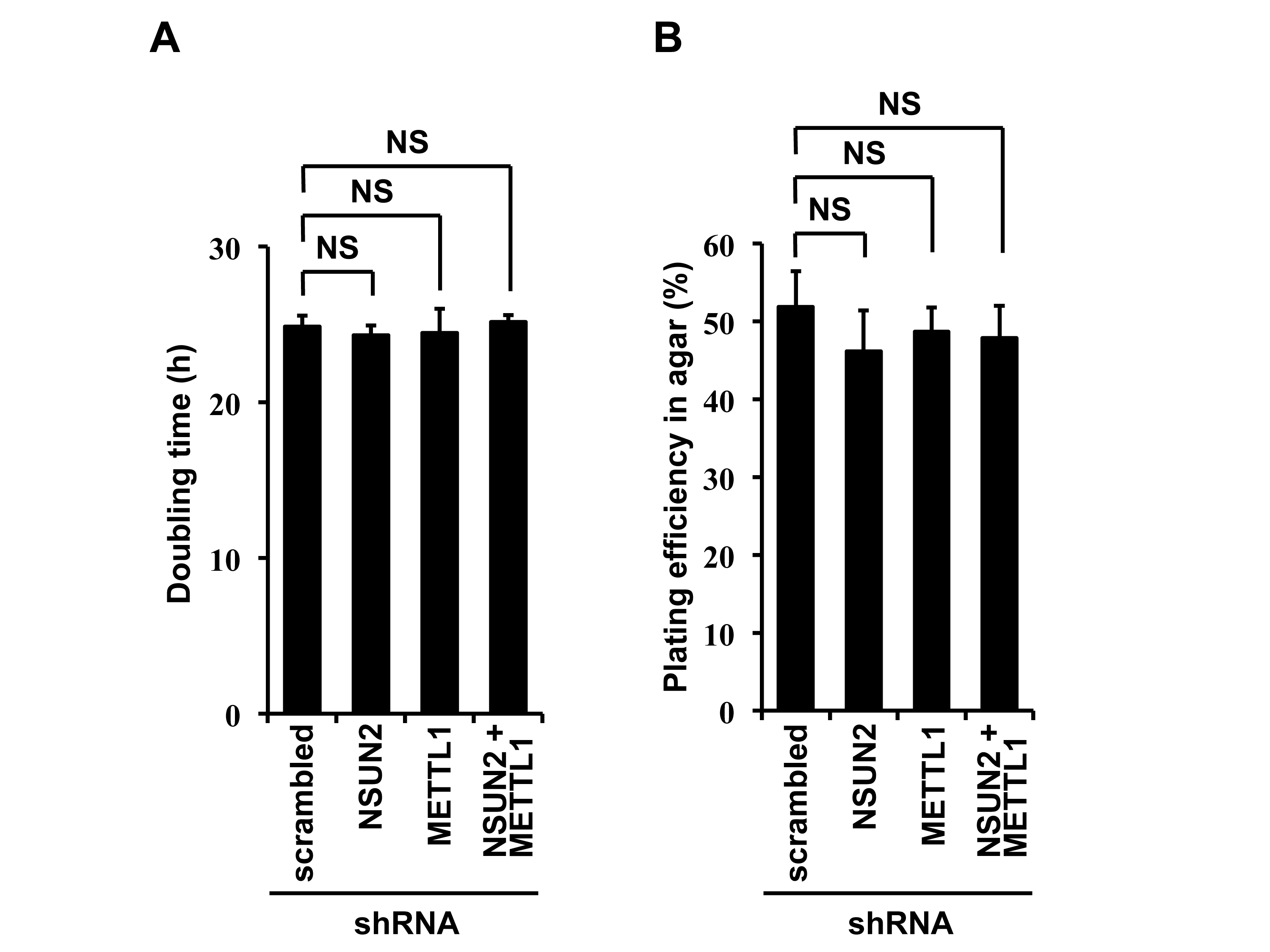

Supplement: Figure S5 — Effects of decreased expression of NSUN2 and METTL1 on cell growth and on anchorage-independent growth in UTR-targeting shRNA-mediated newly established knockdown cells. (A) In vitro doubling times of NSUN2 knockdown cells (lane NSUN2), METTL1 knockdown cells (lane METTL1), NSUN2 and METTL1 knockdown cells (lane NSUN2 + METTL1) and scrambled control vector-transfected cells (lane scrambled). (B) Colony-forming abilities of NSUN2 knockdown cells (lane NSUN2), METTL1 knockdown cells (lane METTL1), NSUN2 and METTL1 knockdown cells (lane NSUN2 + METTL1) and scrambled control vector-transfected cells (lane scrambled) in 0.2% washed agar medium. NS, not significant. (TIF) [file pgen.1004639.s005.tif]

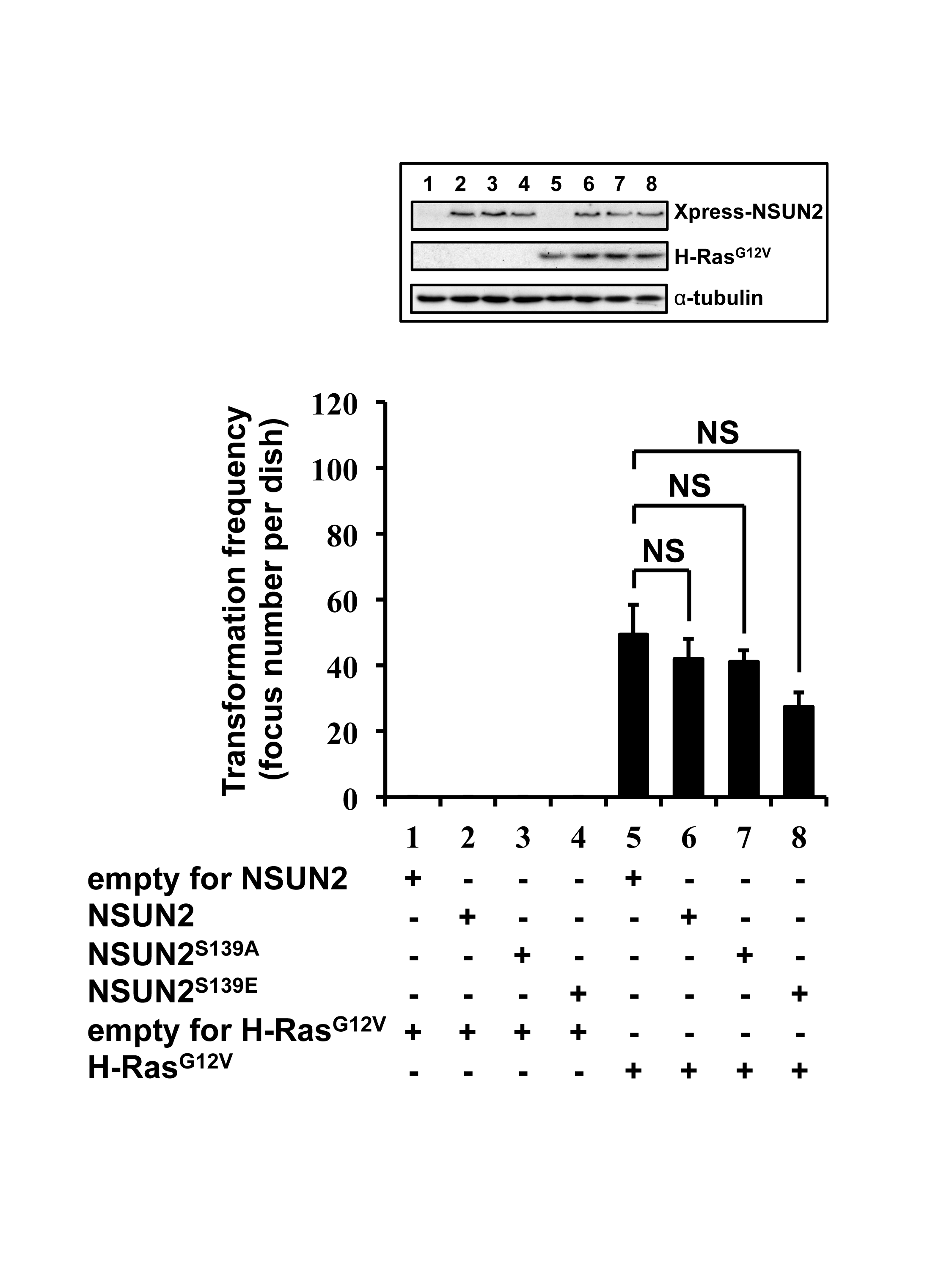

Supplement: Figure S6 — Effects of overexpression of NSUN2, NSUN2S139A, and NSUN2S139E on H-RasG12V-induced cell transformation in vitro. A BALB/c 3T3 A31-1-1 cell transformation assay system was utilized, and quantification of the number of transformed foci was determined using standard criteria [51], [52]. The inset shows an immunoblot of cells transfected with empty vectors (lane 1), NSUN2 (lane 2), NSUN2S139A (lane 3), NSUN2S139E (lane 4), H-RasG12V (lane 5), NSUN2 + H-RasG12V (lane 6), NSUN2S139A + H-RasG12V (lane 7), and NSUN2S139E + H-RasG12V (lane 8). NS, not significant. (TIF) [file pgen.1004639.s006.tif]

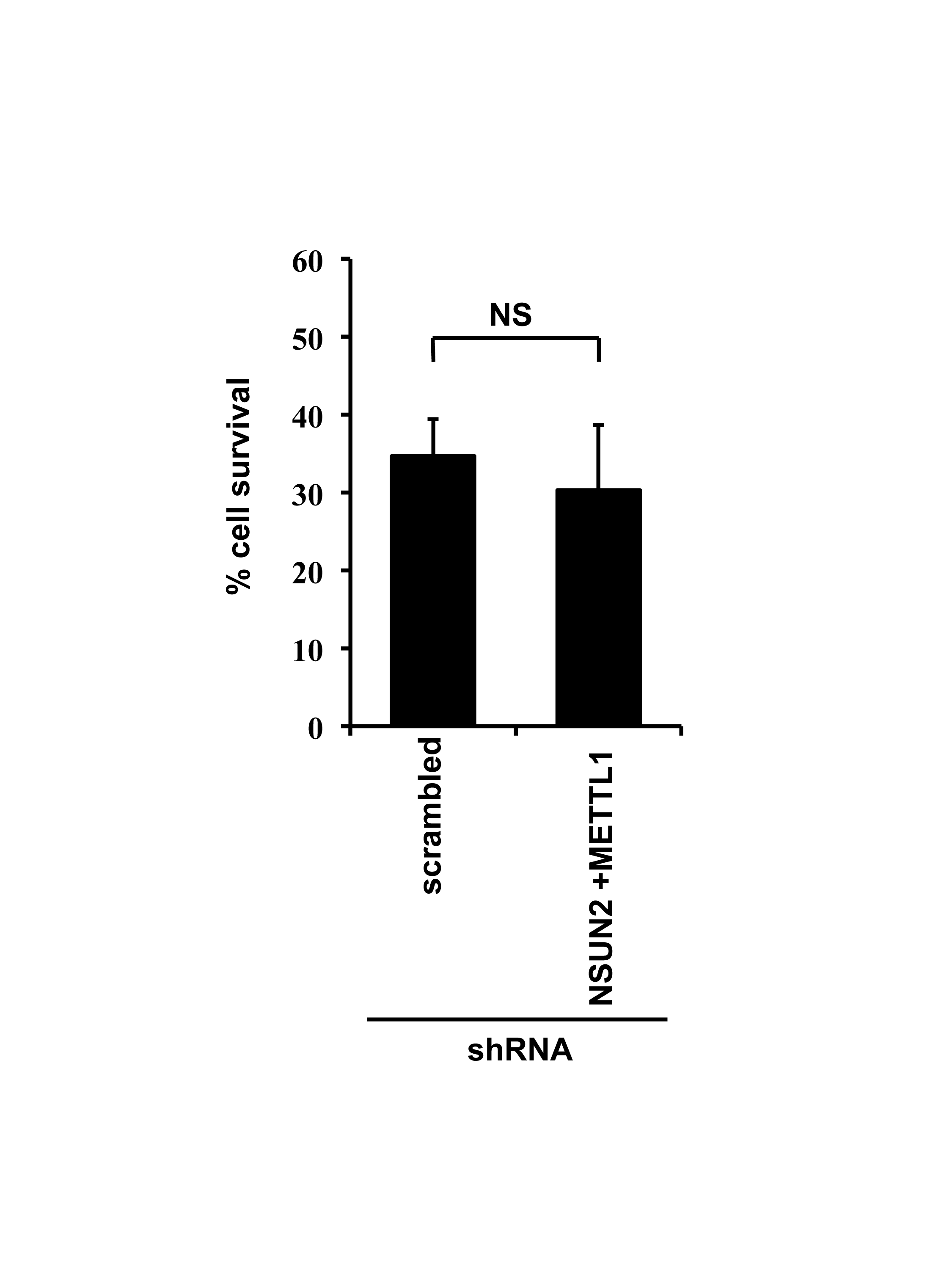

Supplement: Figure S7 — Cell survival in response to 5-FU (IC50 concentrations) under heat stress (43°C for 1.5 h) in NSUN2 and METTL1 knockdown cells (lane NSUN2 + METTL1), and control vector-transfected cells (lane scrambled) with the MTT viability assay. NS, not significant. (TIF) [file pgen.1004639.s007.tif]
